# Single-nucleus RNA sequencing and spatial transcriptomics reveal an immunosuppressive tumor microenvironment related to metastatic dissemination during pancreatic cancer liver metastasis

**DOI:** 10.7150/thno.108925

**Published:** 2025-04-13

**Authors:** Hongsen Liu, Mengting Chen, Bo Hong, Yufei Xiao, Qianming Chen, Yun Qian

**Affiliations:** 1 Stomatology Hospital, School of Stomatology, Zhejiang University School of Medicine, Zhejiang Provincial Clinical Research Center for Oral Diseases, Key Laboratory of Oral Biomedical Research of Zhejiang Province, Cancer Center of Zhejiang University, Engineering Research Center of Oral Biomaterials and Devices of Zhejiang Province, Hangzhou 310000, China.; 2 Department of Clinical Laboratory, Stomatology Hospital, School of Stomatology, Zhejiang University School of Medicine, Zhejiang Provincial Clinical Research Center for Oral Diseases, Key Laboratory of Oral Biomedical Research of Zhejiang Province, Cancer Center of Zhejiang University, Hangzhou 310000, China.; 3 Department of Pathology, The Second Affiliated Hospital, Zhejiang University School of Medicine, Hangzhou 310009, China.; 4 Department of Clinical Laboratory, The Second Affiliated Hospital, Zhejiang University School of Medicine, Hangzhou 310009, China.

**Keywords:** Pancreatic d.uctal adenocarcinoma, Spatial transcriptomics, Single-nucleus RNA sequencing, T_reg_ cells, metastatic dissemination, CITED4

## Abstract

**Background:** Pancreatic ductal adenocarcinoma (PDAC) is a highly aggressive malignancy characterized by early liver metastasis and high mortality. The tumor microenvironment plays a pivotal role in tumor progression; however, the immune microenvironment's involvement in PDAC liver metastasis remains poorly understood.

**Methods:** This study investigates cellular heterogeneity in primary tumor (PT) and liver metastasis (LM) tissues of PDAC using single-nucleus RNA sequencing and spatial transcriptomics. Intra-tumor heterogeneity and cell interactions were elucidated through deconvolution, intercellular signalling, pseudotime analysis, and immune infiltration profiling. The spatial distribution of immune cells was assessed by multiplexed immunofluorescence staining, and prognostic models were developed and validated through immunohistochemistry (IHC). Analyzing the regulatory role of CITED4 in the invasion and metastasis of pancreatic cancer cells through transwell assay and scratch wound healing assay.

**Results:** A total of 62,326 cells were sequenced, with metastatic dissemination cells showing significant upregulation of epithelial-mesenchymal transition (EMT)-related genes during liver metastasis. Spatial transcriptomics revealed the enrichment of metastatic dissemination cells and FOXP3-related T_reg_ cells at the tumor front in PT tissues. In comparison to LM tissues, the tumor front in PT tissues fosters an immunosuppressive microenvironment through the accumulation of T_reg_ cells. Interaction analysis identified the SPP1 pathway as a key promoter of this immunosuppressive environment. Furthermore, prognostic models highlighted CITED4 as critical biomarkers in PDAC. Elevated CITED4 expression is correlated with liver metastasis and poor prognosis in patients with PDAC. siRNA-mediated knockdown of CITED4 suppresses the invasion and metastasis of pancreatic cancer cells.

**Conclusions:** In summary, this study revealed that T_reg_ cell alterations, mediated by metastatic dissemination cells within the immune microenvironment, significantly contribute to PDAC liver metastasis, and that CITED4 enhances the metastatic potential of metastatic dissemination cells.

## Introduction

Pancreatic ductal adenocarcinoma (PDAC) is the most prevalent pancreatic malignancy, with rising incidence and mortality rates, and a dismal five-year survival rate of under 10% 1, 2. PDAC is clinically characterized by early distant metastasis, with over 80% of patients exhibiting metastatic spread at diagnosis. Given the nonspecific or asymptomatic nature of pancreatic cancer symptoms, many patients present with regional or distant metastasis, rendering radical surgery unfeasible for the majority 3. The liver is the predominant site of distant metastasis in PDAC. However, PDAC with liver metastasis shows marked resistance to conventional treatment regimens, and therapeutic options remain severely limited 4, 5. The complexity of the PDAC microenvironment, including intercellular interactions and spatial heterogeneity, plays a critical role in tumor progression and metastasis 6, 7. These factors contribute to tumor evolution and promote metastatic spread. Identifying the key heterogeneous elements and intercellular pathways driving PDAC liver metastasis is essential for risk assessment and prognostic evaluation.

Single-cell RNA sequencing (scRNA-seq) is a high-resolution genomics technique that provides insight into intratumoral heterogeneity and the intricate tumor microenvironment (TME) at the single-cell level, offering advantages over traditional large-scale sequencing methods 8, 9. However, pancreatic tissues, rich in enzymes, pose a challenge for scRNA-seq, as these enzymes are released during tissue homogenization and damage cells, leading to cellular destruction and lysis 10. Single-nucleus RNA sequencing (snRNA-seq), which isolates nuclei from formalin-fixed, paraffin-embedded (FFPE) tissues rather than whole cells, offers a viable alternative to scRNA-seq 11. As transcriptional activity in FFPE tissues is preserved, snRNA-seq maintains transcriptional states without introducing dissociation biases, allowing for accurate identification of distinct cell subpopulations 12. Nevertheless, snRNA-seq does not retain spatial information or in situ intercellular communication networks. Spatial transcriptomics (ST) technology overcomes this limitation by combining gene expression with tissue localization, enabling the identification of transcriptomic changes within specific tissue regions 13. The integration of single-cell and spatial transcriptomics data enables comprehensive studies of PDAC at the single-cell level, facilitating a more detailed understanding of its spatially organized biology 14.

PDAC is characterized by a complex immune microenvironment characterized by intricate immune cell interactions 15. Single-cell RNA sequencing of tumor and adjacent normal pancreatic tissues has revealed significant heterogeneity in immune cell infiltration, with a notable increase in CD8 T cells exhibiting an exhausted phenotype in the advanced stages of the disease 16. Immune cells, including T cells, are often confined to specific regions within the tumor. Multiplex immunohistochemistry-based analysis of immune cell heterogeneity and spatial distribution among patients with PDAC has demonstrated considerable variations in the total leukocyte count and the density of leukocyte subpopulations across different histopathological regions and between patients 17. Various leukocyte subpopulations play pivotal roles in PDAC progression. For instance, radiotherapy in patients with PDAC promotes the infiltration of regulatory T cells (Tregs) while decreasing NK cell recruitment. Targeted inhibition of STAT3 in Tregs enhances NK-mediated immune surveillance 18. The basal-like ductal subtype correlates negatively with CD8 T cell proportions but positively with Treg and immunosuppressive macrophage (Mp-TGFBI) levels 19. Furthermore, IL-10 secreted by CD38+ B cells suppresses NK cell cytotoxicity 20, and IL-1β fosters tumor cell proliferation *via* immune-suppressive B cells 21. Thus, understanding the heterogeneity of leukocyte populations and their interactions within the PDAC immune microenvironment is essential for developing strategies that modulate immune responses.

This study conducted a comprehensive analysis of the tumor microenvironment differences between primary PDAC tumors and their matched liver metastatic counterparts using single-cell RNA sequencing. By reconstructing the evolutionary trajectories of cancer cells, a subpopulation of disseminating PDAC cells was identified. ST analysis revealed that this disseminating cell cluster localizes at the tumor boundary, and further analysis described the interactions between this subpopulation and immune cell subpopulations. Additionally, this study explored biomarkers associated with disseminating tumor cells and successfully developed a prognostic model for patients with PDAC, examining its relationship with immune infiltration. Understanding the key components and interactions within the metastasis-specific microenvironment during PDAC liver metastasis provides a theoretical framework for advancing early detection and treatment strategies for metastatic PDAC.

## Results

### Revealing cellular heterogeneity in PDAC primary tumors (PT) and liver metastatic lesions (LM) by single-nucleus RNA sequencing

**Figure 1:**
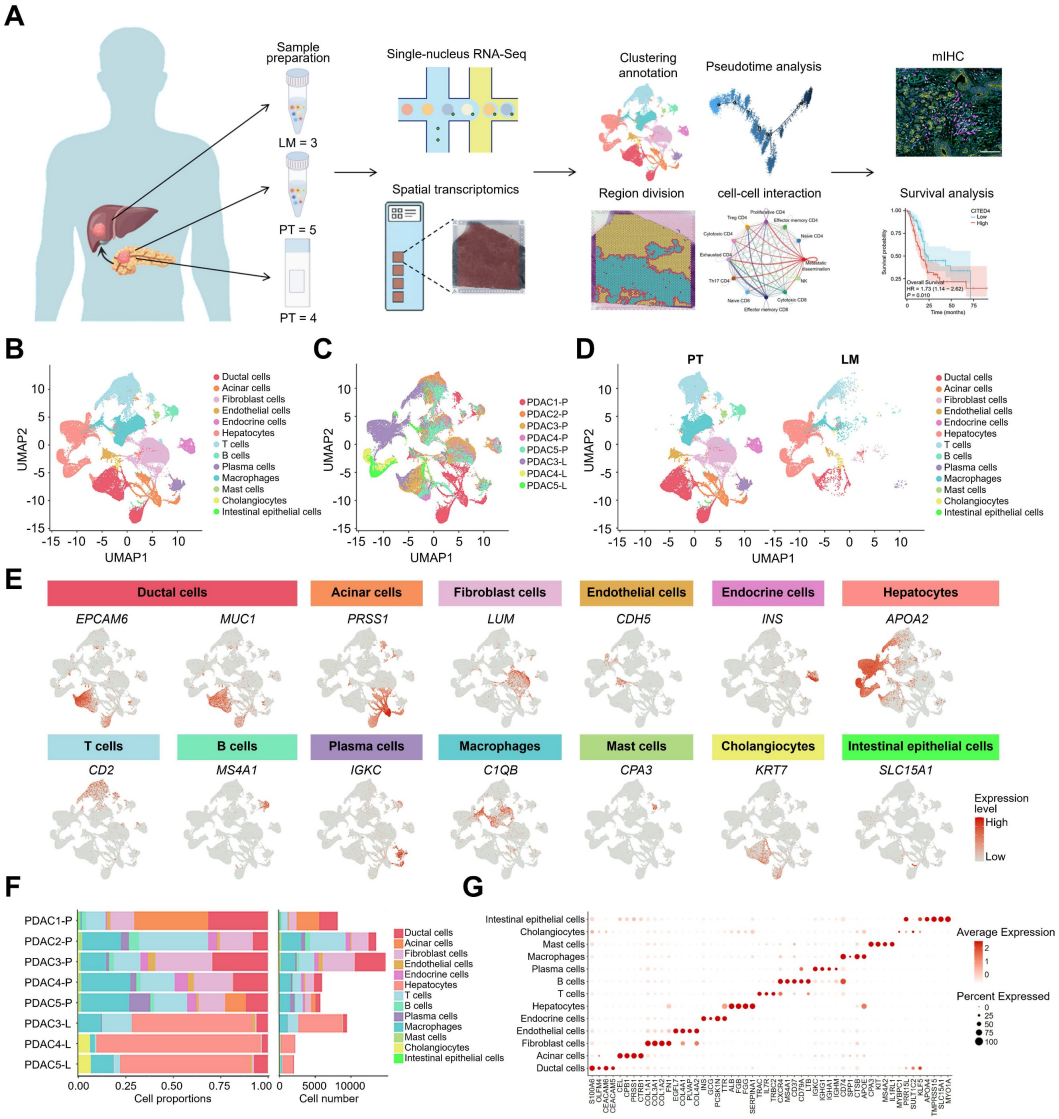
** Landscape of single cell atlas in PDAC. (A)** Workflow of sample acquisition, processing, and analyses from primary and liver metastatic lesions of patients with PDAC. **(B)** UMAP plots of 62,326 high-quality cells from primary tumor (PT) and liver metastatic tumors (LM), showing cell types, colour-coded by inferred cell types. **(C, D)** UMAP plots of the cell atlas showing their sample origins, colour-coded according to their patients **(C)** or tissues **(D)**. **(E)** UMAP plots showing the expression levels of selected marker genes in the cell atlas. **(F)** Bar plots showing the proportions and numbers of each cell type, with each cell type shown in a different colour. **(G)** Dotplot showing the expression levels of major differentially expressed genes in the 13 cell types.

To elucidate the tumor microenvironment (TME) in primary pancreatic tumors (PT) and liver metastases (LM) in patients with PDAC, the 10x Genomics 5' mRNA sequencing method was employed for single-nucleus RNA sequencing of FFPE tissues from five patients with PDAC, including three PTs and their corresponding paired LMs. Additionally, FFPE tissues from four other patients were collected for spatial transcriptomics sequencing, which was integrated with single-cell sequencing data. This approach was further complemented by fluorescence staining and clinical prognostic models to validate the findings (**Figure 1A**). Normalization and dimensionality reduction were performed using Seurat, followed by unsupervised clustering analysis and rigorous quality filtering, resulting in the identification of 62,326 cells for further analysis. Based on marker genes within cell clusters and known cell-specific markers 22-29, the cells were classified into 13 distinct types: ductal cells (EPCAM6, MUC1), acinar cells (PRSS1), fibroblasts (LUM), endothelial cells (CDH5), endocrine cells (INS), hepatocytes (APOA2), T cells (CD2), B cells (MS4A1), plasma cells (IGKC), macrophages (C1QB), mast cells (CPA3), cholangiocytes (KRT7), and intestinal epithelial cells (SLC15A1) (**Figure 1B-E**). To investigate differences in cell type composition between patients with PDAC, the proportion of each cell type in the tissues was calculated. The results indicated that PT tumors were predominantly composed of ductal cells, T cells, B cells, mast cells, and macrophages, while LM lesions showed a higher prevalence of hepatocytes, consistent with the liver's characteristic cellular composition (**Figure 1F**). Notably, the proportions of different cell types varied across patients. Additionally, a dotplot was generated to illustrate the expression levels of differentially expressed genes (DEGs) across the cell types (**Figure 1G**). Ductal cells primarily expressed PDAC-related genes such as S100A6 (Calcyclin) and CEACAM6 (Carcinoembryonic antigen-related cell adhesion molecule 6) 22, 30. Consequently, the ductal cell type was examined to further explore its heterogeneity during PDAC liver metastasis and its regulatory interactions within the TME.

### Activation of inflammatory pathways during metastatic dissemination of malignant ductal cells

**Figure 2:**
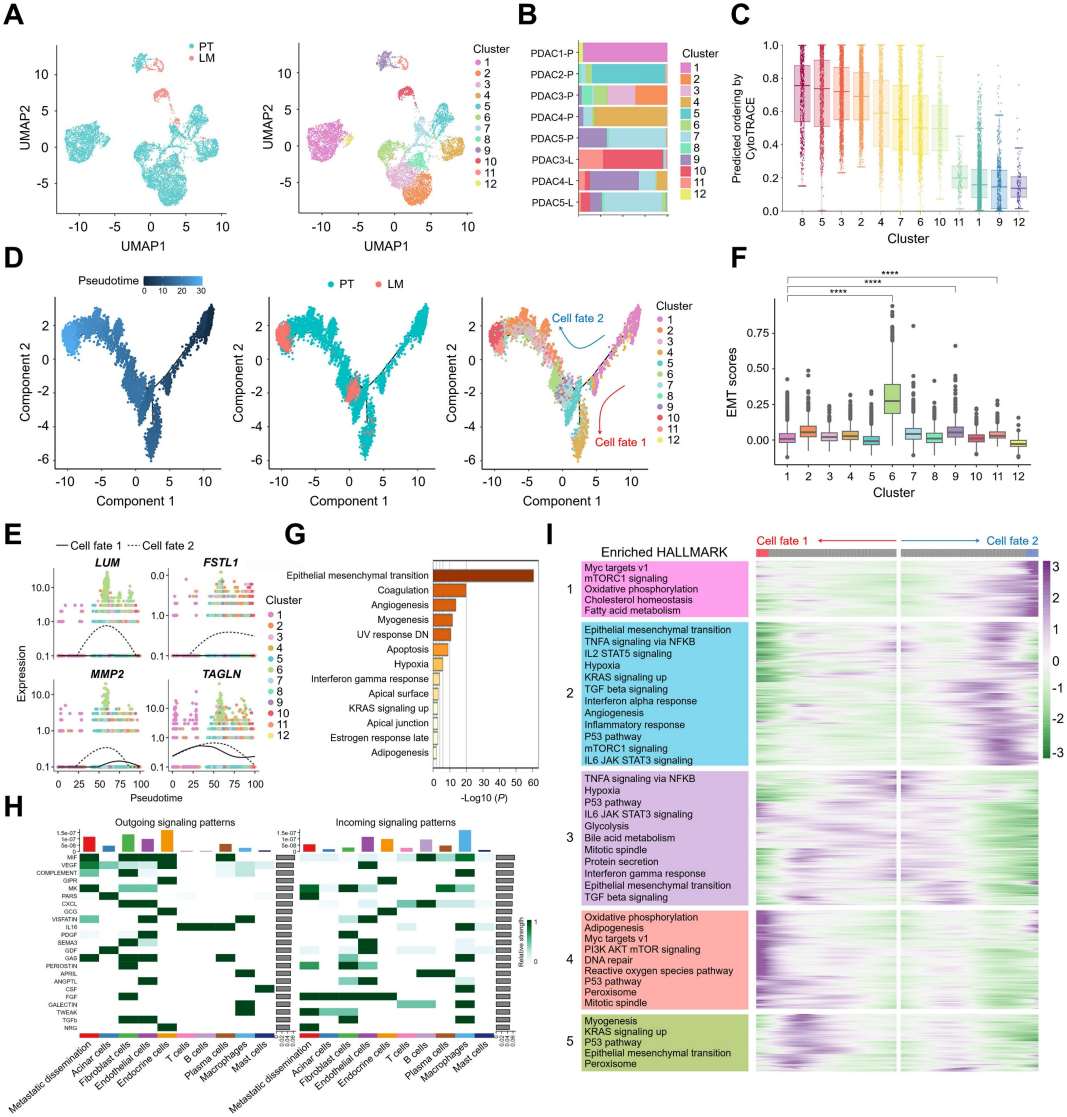
** Transcriptional signatures of metastatic dissemination cells identified by snRNA-seq. (A)** UMAP plot of the ductal cells landscape. Cells were coloured according to their tissues (left) or clusters (right). **(B)** Bar plots showing the cell proportion of each ductal cell subcluster from each sample. **(C)** CytoTRACE predicts the stemness and differentiation potential of each ductal cell subcluster. **(D)** Monocle pseudotime trajectory analysis of ductal cells during the progression process. **(E)** Dot plots of LUM, FSTL1, MMP2, and TAGLN along two cell fates. **(F)** Box plots showing EMT scores for each ductal cell subcluster, statistical testing performed using the t-test (****, p < 0.0001). **(G)** Hallmark pathway enrichment analysis of differentially expressed genes between metastatic dissemination cells and other ductal cell subclusters. **(H)** Heatmap showing the relative strength of the outgoing and incoming signalling pathways in each cell type. **(I)** Hierarchical heatmap and KEGG analysis showing pathways of gene expression patterns of two cell fates across pseudotime.

As PDAC originates from ductal cells, gene expression analysis and subpopulation clustering were performed on ductal cells from PT and LM, identifying 12 distinct ductal cell subpopulations (**Figure 2A & Table S1**). Analysis of cell type proportions in PT and LM revealed that ductal cells in LM were predominantly distributed in ductal cell subpopulations 10 and 11 (**Figure 2B**). We used CytoTRACE 31 to analyze the cell differentiation level between ductal cell subpopulations (**Figure 2C & Figure S1A**). Since subpopulation 1 has a high level of cell differentiation, it suggests that subpopulation 1 is a group of normal ductal cells, and therefore is a clear starting point of the ductal cell evolutionary trajectories. To explore the evolutionary dynamics of ductal cell trajectories during PDAC metastasis, pseudotime analysis of tumor cell subpopulations was conducted using the Monocle method 32, revealing two distinct differentiation trajectories originating from ductal cells in PT, with ductal cells from LM positioned at the terminus of these trajectories (**Figure 2D**). Genes such as LUM, FSTL1, MMP2, and TAGLN were initially upregulated along these trajectories, followed by downregulation (**Figure 2E**). EMT scores analysis revealed significant upregulation of EMT-related genes in subpopulations 6, 9, and 11 compared to subpopulation 1 (p < 0.0001) (**Figure 2F**), which were present in both primary and liver metastatic tissues (**Figure S1B & S1C**). These subpopulations were thus identified as metastatic dissemination cell clusters. Further gene expression changes in metastatic dissemination cells were investigated *via* enrichment analysis of their signature gene sets using the Hallmark database, revealing strong enrichment in EMT, coagulation, and angiogenesis pathways (**Figure 2G**). We further performed differential analysis on the subpopulation 6, 9, 11 of metastatic dissemination cells. In the metastasis-related genes analysis (**Figure S1D**), we found that subpopulation 6 specifically expressed ACTA2, VIM, MMP1, and MMP2, subpopulation 9 specifically expressed CD4, IL32, and VEGFA, subpopulation 11 specifically expressed ANPEP and CDH2. In the Hallmark-related pathway analysis (**Figure S1E**), subpopulation 6 was highly enriched in EMT, reactive oxygen species pathway and PI3K-AKT-mTOR signaling, subpopulation 9 was highly enriched in apical surface, KRAS signaling dn, subpopulation 11 was highly enriched in coagulation, notch signaling. Subpopulation 6 demonstrated strong metastatic potential, and gene enrichment analysis using the Gene Ontology (GO) database revealed significant enrichment in pathways related to collagen-containing extracellular matrix and extracellular matrix organization (**Figure S1F**). To elucidate the crosstalk between metastatic dissemination cells and other tumor micro-environment components, the CellChat method was employed to visualize cell-cell interactions 33. The heatmap in **Figure 2H** illustrates the overall signalling flow between cells, highlighting specifically activated interactions within metastatic dissemination cells, including VEGF and VISFATIN pathways, which are implicated in vascular endothelial cell proliferation (**Figure 2H**). This suggests that communication between metastatic dissemination cells and endothelial cells through these pathways may drive tumor angiogenesis. Metastatic dissemination cells also have strong migration inhibitory factor (MIF) signal communication, we performed immuno-histochemistry (IHC) for MIF using independent pancreatic cancer tissue microarray (TMA) cohorts comprising primary tumors of PDAC and liver metastatic lesions., the analysis revealed strong MIF expression in primary tumor tissues, highlighting its critical role in promoting metastasis (**Figure S1G & S1H**).

Furthermore, cell fate 2 within the differentiation trajectories was notably enriched with metastatic dissemination cells from both PT and LM tumors, indicating a strong association with liver metastasis (**Figure 2D**). To examine gene expression patterns during cancer cell state transitions, a heatmap was used to depict changes in gene expression across five transition states, followed by KEGG enrichment analysis. Notable upregulation followed by downregulation of TNFA signalling *via* NFKB, IL2-STAT5 signalling, and IL6-JAK-STAT3 signalling pathways in cell fate 2 revealed dynamic shifts in inflammation-related pathways during metastasis (**Figure 2I**). Both cell fate 1 and cell fate 2 trajectories demonstrated enrichment in the oxidative phosphorylation pathway, suggesting that these cell fates, particularly tumor cells that have metastasized to the liver, may primarily rely on oxidative phosphorylation for proliferation (**Figure 2I**). In summary, these results indicate that metastatic dissemination cells are enriched in cell fate 2, which is associated with liver metastasis, and exhibit distinct gene expression profiles when compared to non-metastasized and liver-metastasized tumor cells. Additionally, metastatic dissemination cells may exert pro-inflammatory functions, underscoring their potential regulatory role in liver metastasis.

Subsequently, we utilized a published scRNA-seq dataset of pancreatic ductal adenocarcinoma (PDAC) to validate the gene expression patterns of metastatic dissemination cells 23. This dataset comprised primary tumors and liver metastatic lesions. Cells were classified into 9 distinct types: ductal cells, fibroblast cells, endothelial cells, stellate cells, T cells, NK cells, B cells, macrophages, and mast cells (**Figure S2A-C**). Subcluster analysis of ductal cells identified 13 distinct subpopulations (**Figure S2D**). EMT scores analysis revealed significant upregulation of EMT-related genes in subpopulation 11 (**Figure S2E**). Pseudotemporal analysis demonstrated that subpopulation 11 occupied an intermediate position along the trajectory (**Figure S2F**), with genes such as LUM, FSTL1, and CITED4 initially upregulated followed by downregulation along these trajectories (**Figure S2H**). Enrichment analysis of subpopulation 11-specific gene sets using the Hallmark database showed significant enrichment in TNFA signaling via NFKB, EMT and coagulation pathways (**Figure S2I**). Consistent with our findings, re-analysis demonstrates that metastatic dissemination cells possess both metastatic potential and immunoregulatory capacity.

### Immune suppressive landscape of lymphocytes in PDAC primary tumors and liver metastatic lesions

**Figure 3:**
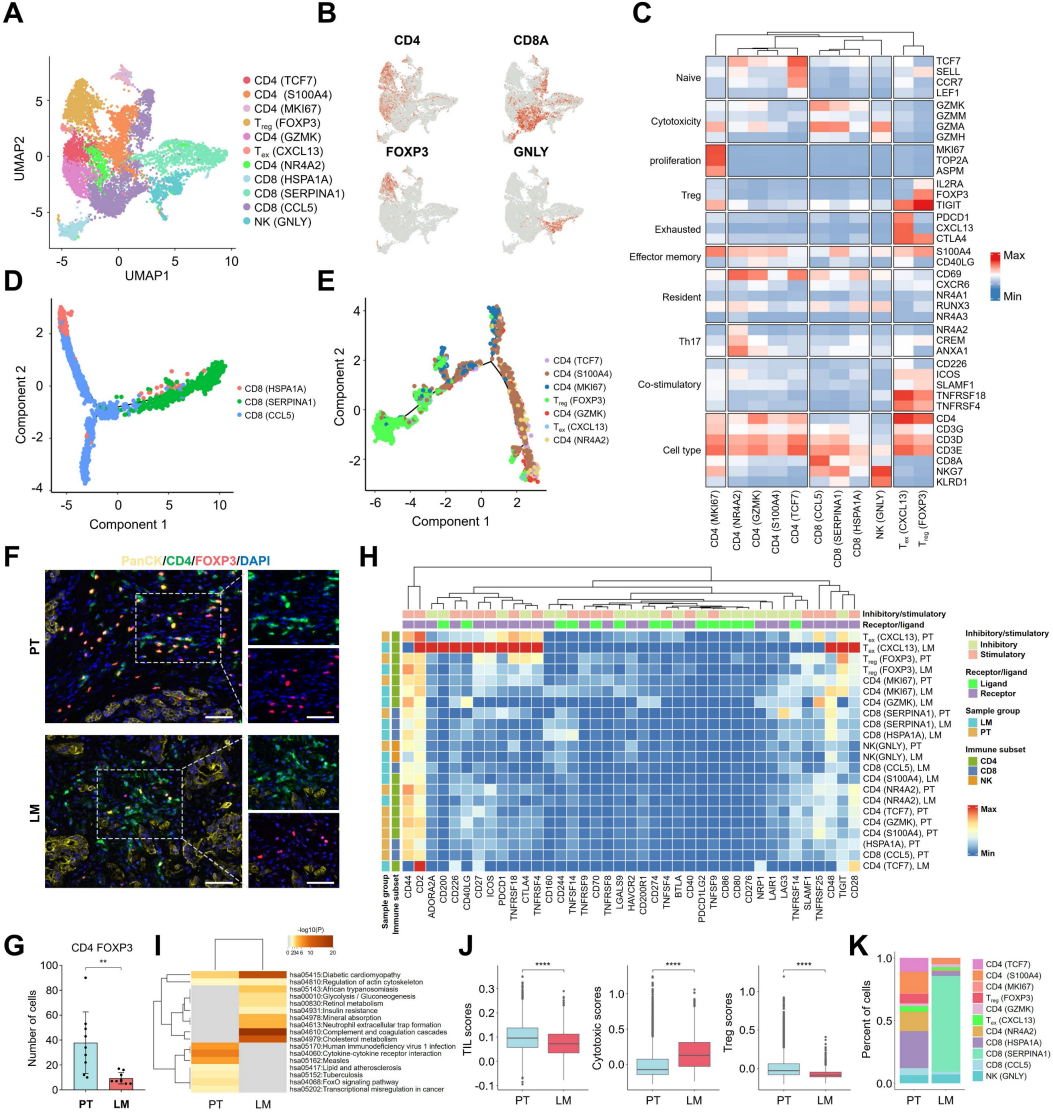
** Transcriptional signatures of lymphoid cells identified by snRNA-seq. (A)** UMAP plot of the lymphoid cell landscape, coloured by subcluster. **(B)** Feature plots showing the normalized expression of specific marker genes in lymphoid cells. **(C)** Heatmap showing the average expression levels of selected genes across 11 lymphoid subclusters. **(D)** Monocle pseudotime trajectory analysis of CD8 cells during the progression process. **(E)** Monocle pseudotime trajectory analysis of CD4 cells during the progression process. **(F)** Immunofluorescence assay for PanCK, CD4, FOXP3, and DAPI in PT and LM samples. Scale bars, 50 µm. **(G)** Statistical analysis of the number of CD4 FOXP3 T cells in PT and LM, statistical testing was performed using the t-test (**, p < 0.01). **(H)** Heatmap of immune checkpoint genes in lymphoid cells, with annotations of receptor or ligand, inhibitory or stimulatory roles. The bars at the top indicate receptor or ligand type, while the left bars indicate sample source and lymphoid cell type annotations. **(I)** KEGG pathway enrichment analysis of differentially expressed genes between CD4 FOXP3 T cells from PT and LM, with a significance threshold of p < 0.05 and log_2_(fold change) ≥ 0.5. **(J)** Box plots showing TIL (tumor-infiltrating lymphocytes), cytotoxic, and Treg scores for lymphoid cells in PT and LM, statistical testing performed using the t-test (****, p < 0.0001). **(K)** Bar plots showing the proportions of 11 lymphoid subclusters in PT and LM.

To investigate the immune environment of primary PDAC tumors and liver metastatic lesions, high-resolution re-clustering of T cells and NK cells was performed to identify distinct subtypes (**Figure 3A**). Based on the transcriptional profiles of key marker genes, including CD4 (TCF7), CD4 (S100A4), CD4 (MKI67), T_reg_ (FOXP3), CD4 (GZMK), T_ex_ (CXCL13), CD4 (NR4A2), CD8 (HSPA1A), CD8 (SERPINA1), CD8 (CCL5), and NK (GNLY), 11 distinct subpopulations were identified (**Figure 3B, C**). T_ex_ cells (Exhausted T cells), characterized by CXCL13 expression, exhibited elevated levels of immune checkpoint proteins such as PDCD1 (PD-1) and CTLA4, while T_reg_ cells positive for FOXP3 showed high expression of IL2RA and TIGIT. Compared to other lymphocyte subsets, T_reg_ and T_ex_ cells displayed higher expression of "co-stimulatory" and "exhaustion" gene signatures (**Figure 3C**). To trace the differentiation trajectories of CD8 T and CD4 T cells, Monocle analysis was used to visualize the cellular differentiation process (**Figure 3D, E**). CD8 (HSPA1A) cells marked the initial stage of CD8 T cell differentiation, branching into CD8 (SERPINA1) and CD8 (CCL5) cells, which expressed cytotoxic markers (**Figure 3D & Figure S3A**). Similarly, CD4 (TCF7) cells represented the starting point of CD4 T cell differentiation, subsequently differentiating into CD4 (S100A4) or CD4 (MKI67) cells. T_reg_ (FOXP3) and T_ex_ (CXCL13) cells, characterized by exhaustion markers, were identified as terminal differentiation states (**Figure 3E & Figure S3B**). Immunofluorescence staining revealed a higher abundance of CD4 FOXP3 cells in PT tissues compared to LM tissues (**Figure 3F, G, K**). Additionally, T_reg_ cells in PT tissues exhibited high expression of immune checkpoint genes such as CD27, ICOS, CTLA4, TNFRSF4, TNFRSF18, and TIGIT (**Figure 3H**). CXCL13+ T_ex_ cells in LM tissues showed elevated levels of immune checkpoint proteins, including PDCD1 (PD-1) (**Figure 3H**). These results suggest that multiple immune checkpoints regulate T_reg_ and T_ex_ cell populations. Differential gene analysis of CD4 FOXP3 cells from PT and LM tissues revealed dysregulation of transcription factors, including FoxO, in CD4 FOXP3 cells from PT tissues (**Figure 3I**). Furthermore, lymphocytes enriched in PT tissues exhibited more pronounced "Tumor Infiltrating Lymphocyte (TIL)" and "Treg" signatures, alongside lower cytotoxicity scores (**Figure 3J**). Collectively, these results suggest that T_reg_ cells in PT tissues play a critical role in the metastatic progression of PDAC.

### Overall landscapes of spatial transcriptomics and intercellular communications of metastatic dissemination cells in the immunological microenvironment

**Figure 4:**
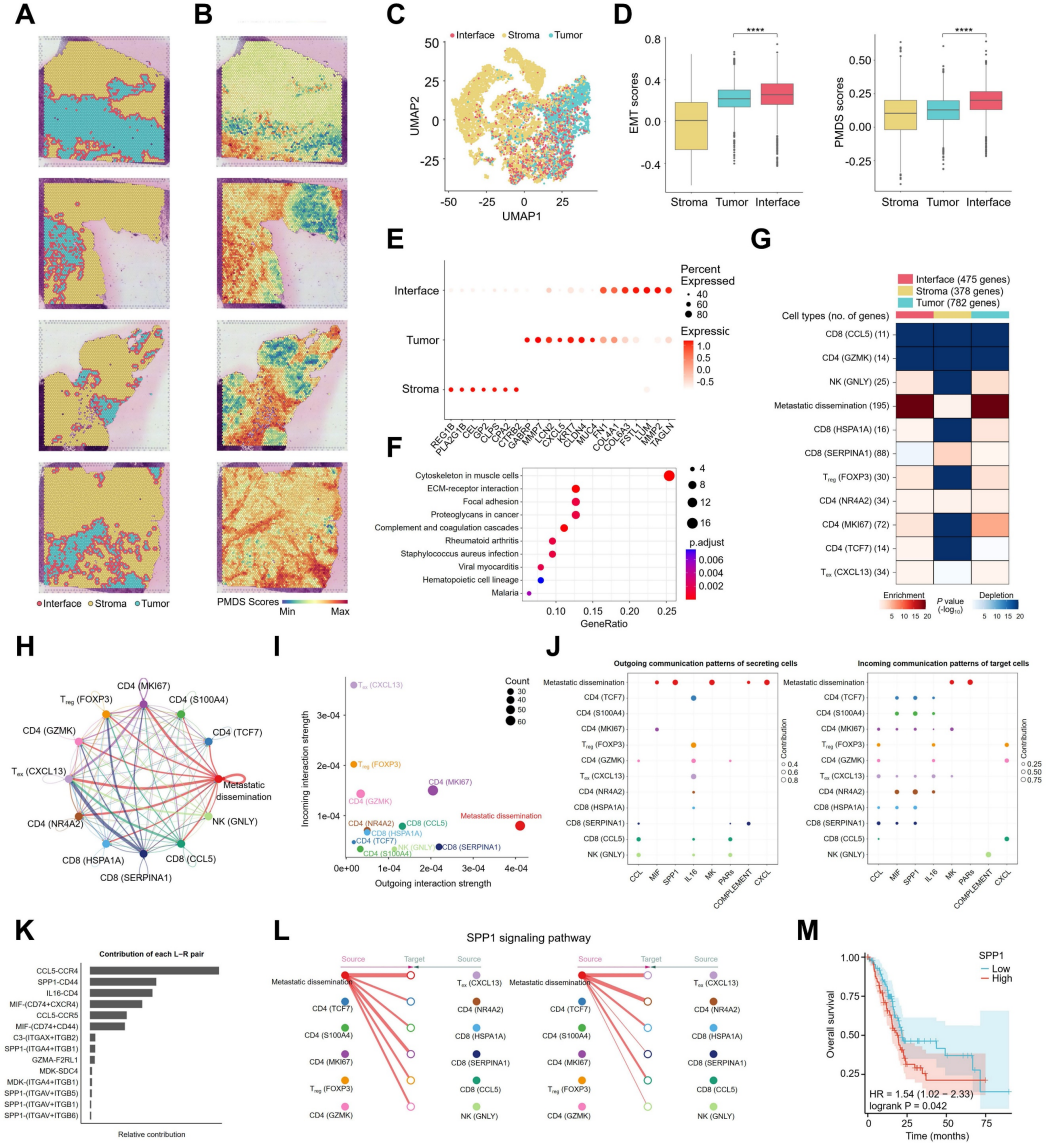
** Spatial features and intercellular ligand-receptor prediction between metastatic dissemination cells and lymphoid cells. (A)** ST spots showing the distribution of tumor, interface, and stroma regions in PDAC primary tumors. **(B)** Spatial feature plots of PMDS in PDAC spatial transcriptomic samples. **(C)** UMAP of unbiased clustering grouped according to tumor, interface, and stroma regions. **(D)** Box plots showing EMT scores and PMDS scores in three regions. Statistical testing was performed using the t-test (****, p < 0.0001). **(E)** Bubble plots showing the expression levels of selected differentially expressed genes across the three regions. **(F)** Bubble plot showing significant KEGG pathways enriched for differentially expressed genes between the interface region and other regions. **(G)** MIA map of overlap between snRNA-seq-defined metastatic dissemination cells, lymphoid cells, and ST-defined tumor, interface, and stroma regions. **(H)** Circle plot showing the strength of intercellular interactions between metastatic dissemination cells and lymphoid cells. (I) Scatter plot showing the relative strength of the outgoing and incoming signalling pathways in metastatic dissemination cells and lymphoid cells. **(J)** Bubble plots showing the specific ligand-receptor pairs of outgoing and incoming signalling pathways in metastatic dissemination cells and lymphoid cells. **(K)** Contribution of each ligand-receptor pair in metastatic dissemination cells and lymphoid cells. **(L)** Hierarchical plot showing the strength of the SPP1 signalling pathway between metastatic dissemination cells and lymphoid cells. (M) Kaplan-Meier curve showing the overall survival of patients with pancreatic adenocarcinoma according to SPP1 expression.

To assess the spatial correlation between metastatic dissemination cells and the immune microenvironment, spatial transcriptomics sequencing was performed on FFPE sections from four patients with PT tissues. Using the SpaCET method 34, PT tissues were categorized into three distinct regions: tumor, interface, and stroma (**Figure 4A**). To further investigate the spatial characteristics of metastatic dissemination cells, UMAP analysis of the ST data revealed clear clustering patterns corresponding to the tumor, interface, and stroma (**Figure 4C**). Pancreatic metastatic dissemination signature (PMDS) scores were developed based on the gene expression profiles of metastatic dissemination cells, and both EMT and PMDS scores were calculated across different tumor regions.

The results indicated that EMT and PMDS scores were significantly higher in the tumor interface compared to the intratumoral region (**Figure 4B, D**). which was consistent with SPOTLight 35 results (**Figure S3C**) and reanalyzed data from a previous study (**Figure S2J**). Differential gene expression analysis across the three regions (**Figure 4E & Table S2**) identified genes such as LUM, FSTL1, MMP2, and TAGLN, which are associated with metastatic dissemination cells, as highly expressed in the interface region. In the tumor region, DEGs included ECM-related genes such as MMP7, LCN2, chemokine CXCL5, and tumor markers CLDN4 and MUC4. Gene enrichment analysis revealed that pathways enriched in the interface region included ECM-receptor interaction, focal adhesion, and complement and coagulation cascades (**Figure 4F**), aligning with the characteristics of metastatic dissemination cells. These results suggest that metastatic dissemination cells are predominantly located at the tumor front. To further investigate the spatial distribution of metastatic dissemination cells in relation to lymphoid cells, data from snRNA-seq and ST were integrated using the MIA method 36. The MIA analysis showed that metastatic dissemination cells, T_reg_, and T_ex_ cells were enriched in both the tumor and interface regions of PT tissues, while cytotoxic CD8 (CCL5) and CD4 (GZMK) cells were absent from these regions (**Figure 4G**), suggesting an immune-suppressive microenvironment at the tumor interface.

The CellChat method was further employed to visualize the interactions between metastatic dissemination cells and lymphoid cells. **Figure 4H** illustrates the interaction strength of signalling pathways between cells, showing that metastatic dissemination cells exhibit robust outgoing interaction strength, whereas T_reg_ and T_ex_ cells display strong incoming interaction strength (**Figure 4I**). Specific ligand-receptor interactions between metastatic dissemination cells and lymphoid cells were investigated, identifying key pairs such as SPP1-CD44 and MIF-(CD74+CXCR4) (**Figure 4J, K**). These results suggest that SPP1 and MIF signaling pathways play a critical role in the metastasis of metastatic dissemination cells. CCL and IL16 signaling pathways also play crucial regulatory roles between immune cells (**Figure S3D**). A detailed analysis of the SPP1 signalling pathway revealed that SPP1 is secreted by metastatic dissemination cells and interacts with all lymphoid cells as target (recipient) cells (**Figure 4L**). Kaplan-Meier survival analysis using TCGA data demonstrated that high SPP1 expression in patients with PDAC correlates with poorer overall survival (OS) (**Figure 4M**). Additionally, we also analyzed the interactions between non-metastatic dissemination cells within ductal cells and lymphocytes. Non-metastatic dissemination cells exhibited strong outgoing interaction strengths (**Figure S3E**), and the specific ligand-receptor interactions between non-metastatic dissemination cells and lymphocytes were primarily mediated by GALECTIN and GDF signaling pathways (**Figure S3F-H**). In summary, an immune atlas at the spatial level was successfully constructed, and the immune networks in different tumor regions were characterized. Moreover, CellChat analysis revealed that metastatic dissemination cells primarily regulate the transformation of lymphoid cells *via* the SPP1 signalling pathway.

### tumor-associated macrophages (TAMs) play an important role in metastatic dissemination microenvironment

Next, we identified 10 distinct subsets of myeloid-derived cells through high-resolution reclustering analysis based on the expression of key marker genes 37, including conventional dendritic cells type 1 (cDC1s), conventional dendritic cells type 2 (cDC2s), myeloid dendritic cells (mDCs), monocytes, lipid-associated TAMs (LA-TAMs), resident-tissue TAMs (RTM-TAMs), pro-angiogenic TAMs (Angio-TAMs), immune regulatory TAMs (Reg-TAMs), neutrophils, and mast cells (**Figure S4A & S4B**). Angio-TAMs may activate anti-tumor immune responses, and they express high levels of co-stimulatory factors (SPP1), proinflammatory cytokines (IL4I1), and lymphocyte migration-related molecules (ITGB7), LA-TAMs represent an immunosuppressive subset of TAMs, characterized by elevated expression of several negative immunoregulatory factors, including FKBP5 and GPNMB (**Figure S4C**). Further MIA analysis revealed that metastatic dissemination cells, monocytes, and TAMs were primarily enriched in tumor and interface regions (**Figure S4D**). KEGG analysis was performed to explore the potential biological functions and associated signaling pathways of each cell type (**Figure S4E**). Angio-TAMs were enriched in pathways related to natural killer cell-mediated cytotoxicity. Monocle differentiation trajectory analysis showed that monocytes occupied the initial stage of cellular differentiation, further branching into LA-TAMs and RTM-TAMs (**Figure S4F**). According to CellChat analysis, metastatic dissemination cells interact with TAMs in the tumor microenvironment via signaling pathways such as MIF (**Figure S4G**). We next examined the anti-inflammatory and pro-inflammatory features of TAM subsets (**Figure S4H**) and found that Angio-TAMs exhibited M1-like pro-inflammatory characteristics, while LA-TAMs and Reg-TAMs displayed M2-like anti-inflammatory phenotypes (**Figure S4I**). Myeloid cells have been reported as critical sources of immune checkpoints in tumors 38; thus, we analyzed the immune checkpoint-related gene scores in TAMs (**Figure S4J & Table S3**). Angio-TAMs showed higher immune activity, whereas LA-TAMs and RTM-TAMs exhibited lower immune activity. Collectively, these data highlight the pivotal role of TAMs in the metastatic dissemination microenvironment.

### Reticular-like cancer-associated fibroblasts (rCAFs) exert immunomodulatory effects in metastatic dissemination microenvironment

Fibroblasts, as the primary component of the tumor stroma, include cancer-associated fibroblasts (CAFs) that play critical roles in the tumor microenvironment (TME) by promoting tumor progression, inflammation, and remodeling the extracellular matrix 39. Based on previously reported phenotypic features of fibroblasts 40, we identified 8 distinct subpopulations: matrix CAFs (mCAFs), inflammatory CAFs (iCAFs), interferon response CAFs (ifnCAFs), reticular-like CAFs (rCAFs), tumor-like CAFs (tCAFs), PI16+ CAFs, SMCs, and Pericytes (**Figure S5A & S5B**). iCAFs exhibit potent pro-inflammatory capabilities and express high levels of pro-inflammatory cytokines CCL2 and IL6 (**Figure S5B**). rCAFs were previously reported in tertiary lymphoid structures (TLS) and surround aggregated immune cells 40, expressing elevated levels of NR2F2 (a transcription factor regulating NF-κB pathways) and TIMP1 (modulating immune cell activity) (**Figure S5C**). Monocle trajectory analysis revealed that mCAFs occupy an early stage in cellular differentiation, further differentiating into iCAFs (**Figure S5D**). KEGG pathway enrichment analysis highlighted distinct signaling pathways in each subpopulation, with rCAFs showing enrichment in antigen processing and presentation (**Figure S5E**). MIA analysis further demonstrated that metastatic dissemination cells, monocytes, and CAFs were primarily enriched in tumor and interface regions (**Figure S5F**). CellChat analysis indicated that metastatic dissemination cells interact with CAFs in the TME via pathways including MIF and SPP1 (**Figure S5G-I**). Collectively, these data suggest that rCAFs exert immunomodulatory functions in the metastatic dissemination microenvironment.

### The MIF signaling pathway plays a crucial regulatory role in liver metastasis of metastatic dissemination cells

B cells, as a critical component of adaptive immunity, play multifaceted roles in human cancers 41. We performed re-clustering analysis of B cells in the tumor microenvironment and identified four distinct subsets based on the expression of key marker genes: naive B cells, memory B cells, IGA+ plasma cells, and IGG+ plasma cells** (Figure S6A & S6B)**. MIA analysis revealed that metastatic dissemination cells and B cell subsets were enriched in both tumor and interface regions** (Figure S6C)**. Monocle differentiation trajectory analysis showed that naive B cells occupy the initial stage of cellular differentiation, further diverging into IGA+ plasma cells and IGG+ plasma cells** (Figure S6D)**. Immune-activating genes, such as IL4R*,* CXCR5*,* CD74*,* and CD40*,* were significantly downregulated along the progression trajectory** (Figure S6E)**. TLS play a crucial role in the tumor immune response, characterized by B cell structures surrounded by T cells 42. Multifluorescent staining of pancreatic cancer tissues was conducted using antibodies against CD4 (white), CD8 (light blue), CD20 (pink), FOXP3 (red), VIM (green), and panCK (yellow), along with 4',6-diamidino-2-phenylindole, dihydrochloride (DAPI) (Figure S6F). We observed that CD4 T cells, CD8 T cells, and fibroblast cells surround CD20 B cells, with a significant presence of FOXP3-Tregs cells around TLS. According to the analysis by CellChat, we found that APRIL secreted by Reg-TAMs has a regulatory effect on B cells, the MIF signaling pathway is crucial for the regulation of macrophages, B cells, and T cells by metastatic dissemination cells (Figure S6G). By integrating these data, we provide insights into the regulatory mechanisms of the tumor microenvironment during the metastasis of metastatic dissemination cells from the perspective of cellular interactions.

### Construction and validation of a metastatic dissemination-related prognostic model for pancreatic adenocarcinoma

**Figure 5:**
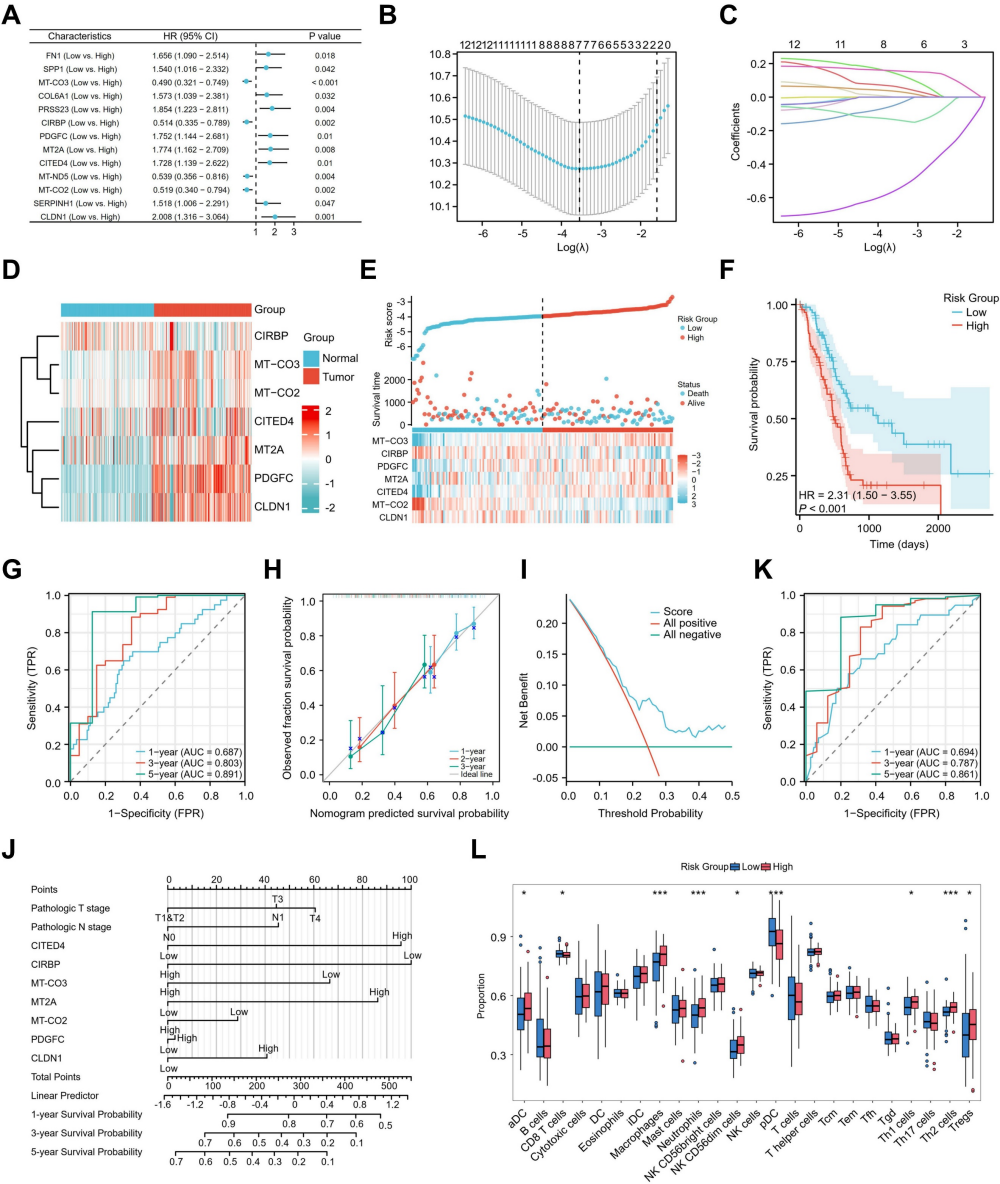
** Identification of a metastatic dissemination-related signatures to predict prognosis and immune landscape in pancreatic adenocarcinoma. (A)** Univariate Cox regression analysis for 13 MDRGs significantly associated with OS. **(B, C)** LASSO regression analysis identified OS-related candidate prognostic genes. **(D)** The heatmap revealed differential expression patterns of seven key MDRGs between normal and tumor tissues. **(E)** Risk score, survival status, and heatmap of the expression levels of the 7 candidate prognostic MDRGs in patients with pancreatic adenocarcinoma. (F) Kaplan-Meier survival analysis for OS between the high- and low-risk groups. **(G)** ROC curves of the prognostic model for predicting 1-, 3-, and 5-year survival rates. (H) Calibration curve of the prognostic model. **(I)** Decision curve analysis (DCA) for the evaluation of the prognostic model. **(J)** Clinical prognostic nomogram model for pancreatic adenocarcinoma. **(K)** ROC curves of the nomogram. **(L)** Boxplot showing the ssGSEA scores of 24 immune cell subsets in high- and low-risk groups.

The potential association between genes expressed by metastatic dissemination cells and patient prognosis was further assessed. A univariate Cox regression analysis was first conducted using TCGA data of pancreatic adenocarcinoma, identifying metastatic dissemination-related genes (MDRGs) associated with OS and disease specific survival (DSS) in patients with pancreatic adenocarcinoma (**Figure 5A & Figure S7B**). Subsequently, a prognostic model was constructed using the LASSO algorithm based on these MDRGs, ultimately selecting seven key MDRGs (MT-CO3, CIRBP, PDGFC, MT2A, CITED4, MT-CO2, and CLDN1) for OS-based model development (**Figure 5B, C & Figure S7A**), and seven key MDRGs (CLDN1, CITED4, MT2A, PDGFC, CIRBP, MT-CO3, and CCN1) for DSS-based model development (**Figure S7C & S7D**). Among the seven key MDRGs, CIRBP was downregulated in tumor tissues, while the other six genes were upregulated in tumor tissues (**Figure 5D**).

**Figure 5E & Figure S7E** show the risk score for each patient with pancreatic adenocarcinoma, patients classified into high-risk and low-risk groups based on their scores. The survival time distribution indicated that as the risk score increased, survival time decreased, and mortality increased. Kaplan-Meier analysis revealed that patients with high-risk scores had significantly lower OS and DSS than those with low-risk scores, indicating a poorer prognosis for the high-risk group (**Figure 5F & Figure S7F**). The model's effectiveness was further evaluated through ROC curve analysis, showing AUC values of 0.687, 0.803, and 0.891 for 1, 3, and 5 years, respectively (**Figure 5G & Figure S7G**). Additionally, the calibration curve demonstrated good predictive accuracy (**Figure 5H & Figure S7H**), while the DCA curve confirmed the model's strong clinical applicability (**Figure 5I & Figure S7I**). To improve clinical utility, a nomogram incorporating clinical parameters was constructed for patients with pancreatic adenocarcinoma (**Figure 5J**). The nomogram generated AUC values for 1-year, 3-year, and 5-year OS of 0.694, 0.787, and 0.861, respectively (**Figure 5K**). To investigate the relationship between the prognostic model and the immune microenvironment, ssGSEA and xCell was used to calculate immune cell infiltration scores in pancreatic adenocarcinoma patients. The results indicated significantly higher infiltration of Treg cells in the high-risk group, while the low-risk group exhibited increased levels of CD8 T cells (**Figure 5L & Figure S8A**), suggesting an immunosuppressive microenvironment in the high-risk group. Subsequent analysis of the correlation between the seven MDRGs and immune infiltration revealed that CITED4, MT-CO2, and MT-CO3 exhibited a positive correlation with Treg infiltration (**Figure S7J**). Immunofluorescence staining revealed that CD4 FOXP3 cell numbers in PT tissues from patients with late-stage pancreatic cancer were significantly higher than those in patients with early-stage pancreatic cancer (**Figure S8B**). In summary, the analysis demonstrates that this prognostic risk model offers excellent predictive performance for OS and DSS in patients with pancreatic adenocarcinoma.

### The expression of CITED4 promotes liver metastasis of pancreatic adenocarcinoma

**Figure 6:**
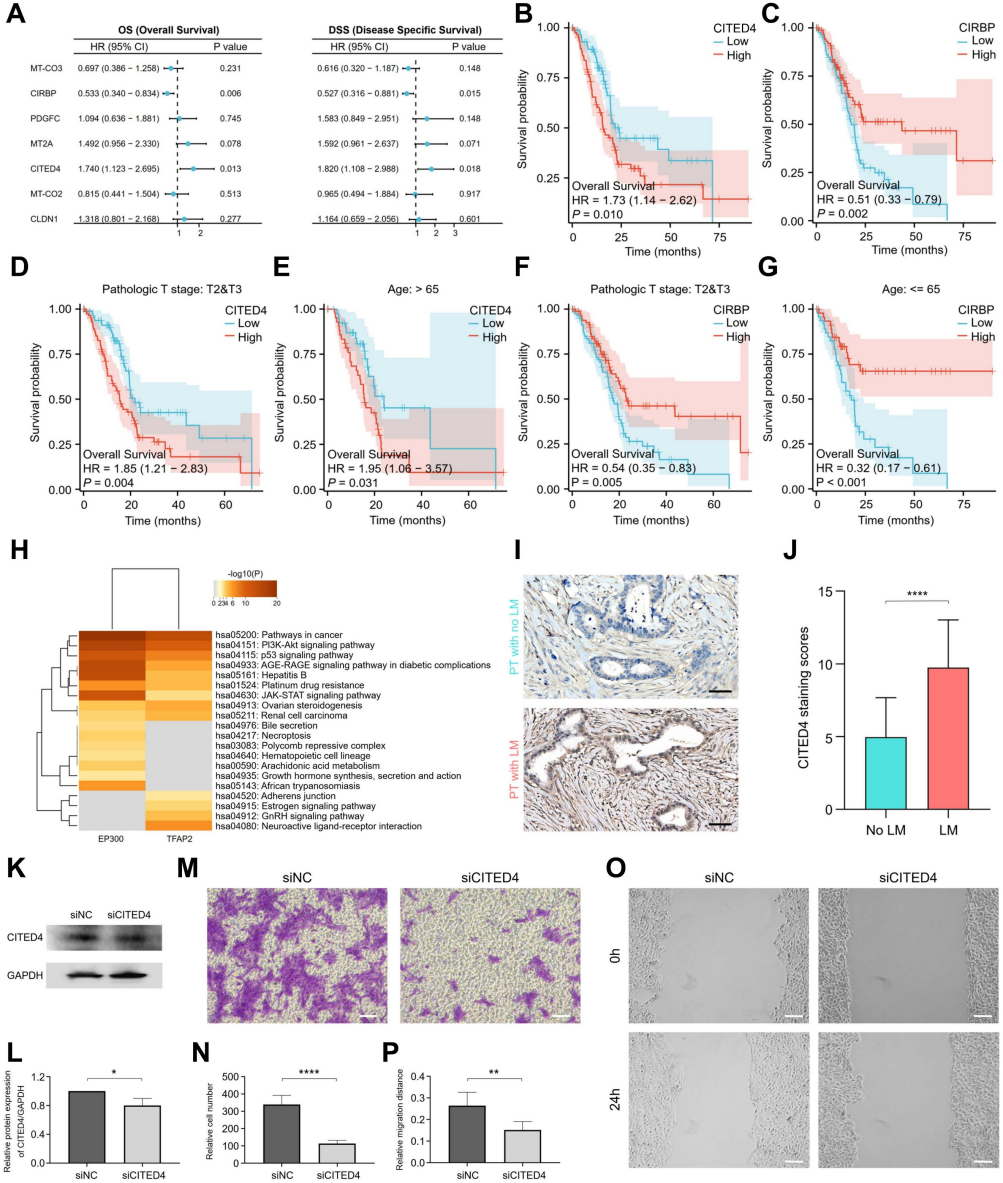
** Prognostic value of CITED4 expression in patients with pancreatic adenocarcinoma. (A)** Multivariate Cox regression analysis of 7 candidate prognostic MDRGs associated with OS and DSS. **(B, C)** Kaplan-Meier curves showing OS of patients with pancreatic adenocarcinoma according to CITED4 expression **(B)** or CIRBP expression **(C)**. **(D, E)** KM survival curves of patients in high- and low CITED4 expression groups stratified by stage **(D)** and age** (E)** subgroups. **(F, G)** KM survival curves of patients in high- and low CIRBP expression groups stratified by stage** (F)** and age** (G)** subgroups. **(H)** KEGG enrichment analysis for target genes of EP300 and TFAP2. **(I)** Immunohistochemistry detection of CITED4 expression in pancreatic adenocarcinoma tissues with or without liver metastasis. Scale bars, 50 µm. **(J)** Quantified staining scores of CITED4 in **(I)**, with statistical testing by t-test (****, p < 0.0001). **(K)** L3.6p1 cells were transiently transfected with siNC and siCITED4, and the protein expression level of CITED4 was detected by western blot. **(L)** Densitometric quantification of CITED4 protein expression relative to GAPDH, statistical testing was performed using the t-test (*, p < 0.05). **(M)** Transwell assay assessing cell migration in response to siCITED4. Scale bar, 100 µm. **(N)** Quantification of migrated cells per field, statistical testing was performed using the t-test (****, p < 0.0001). **(O)** Scratch wound healing assay evaluating cell invasion in L3.6p1 cells after siCITED4. Scale bar, 100 µm. **(P)** Quantification of wound closure rate, statistical testing was performed using the t-test (**, p < 0.01).

Multivariate Cox regression analysis of the seven candidate prognostic MDRGs identified CITED4 and CIRBP as independent prognostic factors for both OS and disease-specific survival (DSS) in patients with pancreatic adenocarcinoma (**Figure 6A**). Kaplan-Meier survival analysis indicated that high CITED4 expression (**Figure 6B**) and low CIRBP expression (**Figure 6C**) were associated with poorer overall survival, disease-specific survival (**Figure S8C**), and progress free interval (**Figure S8D**) outcomes. Prognostic assessment of CITED4 and CIRBP expression, stratified by clinical stage and age, revealed that high CITED4 expression correlated with significantly worse survival in the T2&T3 and age >65 subgroups (**Figure 6D, E**), while low CIRBP expression predicted worse survival in the T2&T3 and age ≤65 subgroups (**Figure 6F, G**). CITED4 expression was specifically enriched in both ductal cells (**Figure S8E**) and metastatic dissemination cells (**Figure S2G & S7A**). CITED4, a member of the CBP/p300-interacting transactivator with a glutamic acid- and aspartic acid-rich tail (CITED) family (CITED1, CITED2, CITED4), interacts with CBP/p300 and the transcription factor TFAP2 to regulate TFAP2 transcription. Emerging evidence indicates that CITED4 plays a pivotal role in cytokine-induced cellular proliferation, differentiation, and tumorigenesis 43. Target genes interacting with EP300 and TFAP2 were analyzed using the TRRUST database 44. KEGG functional annotation revealed that these target genes are predominantly involved in key pathways such as the PI3K-AKT signalling pathway, p53 signalling pathway, and JAK-STAT signalling pathway (**Figure 6H**), highlighting the regulatory role of CITED4 in promoting cancer progression. To assess the potential of CITED4 expression as a prognostic marker, IHC analysis was performed on pancreatic cancer tissues. The results showed elevated CITED4 expression levels in patients with liver metastasis (**Figure 6I, J**). Patients were categorized into two groups based on CITED4 expression, with those exhibiting high CITED4 expression having a higher M stage and more advanced clinicopathological stage (p < 0.0001, **Table 1**). To investigate the relationship between CITED4 and the immune microenvironment, we utilized CIBERSORT to calculate immune cell infiltration scores in pancreatic adenocarcinoma patients stratified by high versus low CITED4 expression. The results revealed a significant increase in Treg cell infiltration within the CITED4 high-expression group compared to the low-expression cohort (**Figure S8F**). To explore the relationship between CITED4 and immunogenic properties, we stratified ductal cells into CITED4-positive and CITED4-negative groups based on CITED4 expression levels (**Figure S8G**). Immunogenic cell death (ICD), which triggers anti-tumor immune responses, was evaluated between these groups 45. The analysis revealed that the CITED4-positive group exhibited significantly lower immunogenicity compared to the CITED4-negative group (**Figure S8H & Table S4**). The migration and scratch wound healing ability of L3.6p1 cells with CITED4 expression suppressed by siRNA were significantly decreased (**Figure 6K-O**). **Figure 5E** stratified pancreatic adenocarcinoma patients into high-risk and low-risk groups based on CITED4 expression profiles. T-cell immune responses-related ICOSLG showed significantly lower expression in the high-risk group, while immune escape-related VTCN1 exhibited elevated expression (**Figure S8I**). Importantly, CITED4 expression negatively correlated with ICOSLG (**Figure S8J**) but positively correlated with VTCN1 (**Figure S8K**). Collectively, these findings suggest that CITED4 contributes to the formation of immunosuppressive microenvironments by suppressing pro-immune pathways while enhancing immunosuppressive mechanisms. These results suggest that CITED4 expression serves as a valuable prognostic indicator in pancreatic cancer.

## Discussion

The metastatic spread of tumor cells remains a leading cause of patient mortality 46. Metastatic dissemination cells, originating from epithelial tissues, possess the ability to detach from the primary tumor and spread to distant organs *via* the bloodstream or lymphatic system. These cells exhibit marked molecular heterogeneity when compared to their primary tumor counterparts 47. The survival and metastatic potential of these cells are significantly influenced by the tumor microenvironment, with immune evasion or suppression playing a pivotal role in their dissemination 48, 49. Consequently, exploring the interactions between metastatic dissemination cells and the immune microenvironment is essential for understanding the mechanisms driving tumor metastasis and for the development of novel therapeutic approaches. In this study, single-cell RNA sequencing was employed to uncover the extensive cellular heterogeneity within PDAC primary tumors and liver metastatic lesions. Combining CNV analysis with the Monocle method enabled the identification of metastatic dissemination cells within ductal cell subpopulations, revealing their involvement in regulating inflammation-related processes. A prognostic model was constructed, identifying CITED4 expression in metastatic dissemination cells as a potential biomarker for predicting clinical outcomes. Furthermore, the identification of T cell and NK cell subtypes, coupled with CellChat analysis, highlighted key signalling pathways between metastatic dissemination cells and immune cells, particularly the SPP1 pathway, which plays a pivotal role in modulating immune processes.

The complex tumor microenvironment significantly contributes to the resistance of PDAC to immunotherapies 50. In this study, multi-colour fluorescent staining revealed a notable increase in CD4 T cells exhibiting exhaustion characteristics surrounding tumor cells in patients with advanced pancreatic cancer (**Figure S8B**). This observation is consistent with recent research utilizing cytometry by time-of-flight (CyTOF), multiplex fluorescent immunohistochemistry (mIHC), and single-cell RNA sequencing, which highlighted the heterogeneity of cytotoxic T cells within the PDAC tumor microenvironment, where the expression of exhaustion markers correlates with tumor malignancy 16. T_reg_ cells are critical for regulating excessive immune activation and maintaining immune homeostasis 51; however, the interaction between T_reg_ cells and metastatic dissemination cells during PDAC progression and metastasis remains poorly understood. Studies have shown that T_reg_ cell enrichment in the liver metastatic microenvironment suppresses anti-tumor immunity 23. Following the construction of a prognostic model for metastatic dissemination cells, a significant increase in T_reg_ cell infiltration was observed in the high-risk group. Patients with high CITED4 levels exhibited significant liver metastasis and a more advanced clinicopathological stage (**Table 1**). Additionally, higher levels of T_reg_ cells were found in the primary tumor tissue, expressing elevated levels of costimulatory genes associated with immune checkpoints (CD27, ICOS, TNFRSF4, and TNFRSF18) and exhaustion markers (CTLA4 and TIGIT). While T_ex_ cells were also abundant in the primary tumor tissue, the expression of exhaustion genes (PDCD1, CTLA4, and TIGIT) was notably higher in liver metastasis (LM) tissue, suggesting that T cell functional exhaustion in the metastatic microenvironment contributes to immune evasion. Spatial transcriptome analysis revealed that metastatic dissemination cells colocalized with T_reg_ and T_ex_ cells at the interface and within tumor regions, metastatic dissemination cells were positively correlated with Treg cell infiltration (**Figure S8F**), with strong output signals from metastatic dissemination cells that were efficiently received by T_reg_ and T_ex_ cells. Among these interactions, the SPP1-CD44 ligand-receptor pair emerged as a key mechanism by which metastatic dissemination cells modulate immune cell function.

TAMs play a dual role in the tumor metastatic immune microenvironment: they not only secrete pro-angiogenic and chemotactic factors to promote metastasis, but also suppress T-cell activity via PD-L1/IL-10 and synergize with Tregs to establish immunosuppressive networks 37. Our findings reveal that LA-TAMs exhibit a unique M2-like polarization and anti-inflammatory signature, distinguished by robust upregulation of immunosuppressive effectors FKBP5 and GPNMB (**Figure S4C**). Functional crosstalk between metastatic dissemination cells and LA-TAMs within the tumor microenvironment was mediated through MIF signaling (**Figure S4G**), underscoring the critical involvement of LA-TAMs in shaping the pro-metastatic immunosuppressive microenvironment.

The reticular-like CAFs (rCAFs), a recently identified fibroblast subpopulation with established roles in organizing tertiary lymphoid structures (TLSs), may play a pivotal role in regulating immune cell trafficking and activation within the tumor microenvironment 40. Transcriptomic profiling reveals their unique immunoregulation ability, marked by NR2F2 and TIMP1. Multifluorescent immunohistochemistry and functional studies demonstrate that rCAFs may exhibit enhanced antigen processing/presentation capabilities (**Figure S5E & S6F**), suggesting a previously unrecognized role in adaptive immune within metastatic niches. Regulatory networks showing that CSF and TGFb signaling emerges as a central axis coordinating TAMs responses to rCAFs (**Figure S6G**). These findings collectively provide novel insights into the immunomodulatory functions of rCAFs within the metastatic TME, highlighting their previously unrecognized roles in shaping the immune landscape. Furthermore, this study elucidates the intricate cross-talk between stromal and immune compartments in the metastatic TME, underscoring their dynamic interactions. These findings provide a theoretical foundation for developing immunotherapeutic strategies targeting TME.

Our study still has several limitations. First, the cell types identified by snRNA-seq were based on a relatively small sample size, and the cell types identified in LM did not completely match those in PT, limiting the systematic analysis of differences in all cell types between PT and LM. Second, although we successfully constructed a prognostic model of the immunosuppressive microenvironment in pancreatic cancer patients, further clarification of the model's value using clinical data related to immunotherapy is still needed for its clinical application and promotion. Furthermore, while our study provided correlation analysis between metastatic dissemination cells and immune cells, further biological experiments are required to validate the molecular mechanisms underlying the regulation of immune cell exhaustion by metastatic dissemination cells.

## Conclusions

In conclusion, our data highlight the substantial heterogeneity of ductal and immune cells in both primary and metastatic PDAC tumors. The prognostic model targeting metastatic dissemination cells demonstrates promising predictive performance. Furthermore, this study identified immune microenvironment alterations during liver metastasis of PDAC and elucidated the interactions between tumor cells and T_reg_ and T_ex_ cells during early dissemination, and the regulatory mechanism by which CITED4 promotes liver metastasis of pancreatic cancer. These findings lay a solid foundation for the development of immunotherapeutic strategies targeting liver metastasis in PDAC, offering potential prognostic and therapeutic insights.

## Materials and methods

### Samples collection and preparation

Tissue samples were collected from five patients (two primary tumors without liver metastases, three primary tumors with liver metastases and three paired liver metastatic lesions) diagnosed as having PDAC for snRNA-seq and four patients diagnosed as having moderately differentiated PDAC for ST sequencing at the Department of Hepatobiliary and Pancreatic Surgery of the Second Affiliated Hospital of Zhejiang University School of Medicine, Zhejiang, China. A senior pathologist rinsed the tissue samples with phosphate-buffered saline (PBS) and fixed them in 10% formalin for 24 h. Following fixation, the samples were washed thoroughly with running water, dehydrated through a graded alcohol series, and cleared with xylene. The tissues were then embedded in paraffin, resulting in FFPE tissue blocks.

### Single-nucleus RNA sequencing library construction

First, 25 or 50 µm sections were prepared from the FFPE blocks, deparaffinized using Xylene (10 min each), and sequentially washed with 100%, 70%, and 50% ethanol, followed by PBS. The tissue was dissociated using the Dissoci Enzyme Mix, employing a 1.5 mL pellet pestle. After filtration and centrifugation, the resulting cells were resuspended in a Tissue Resuspension Buffer. The dissociation process was performed using the gentleMACS Octo Dissociator in a gentleMACS C Tube. The cell suspension was filtered, centrifuged, and barcoded using Chromium Fixed Profiling Reagent Kits per the manufacturer's instructions. DNA libraries were constructed and sequenced on an Illumina NovaSeq6000 to generate 150 bp paired-end reads.

### Data processing of single-nucleus RNA sequencing

The 10× libraries were mapped to the GRCh38 human reference genome using Cell Ranger software (v3.1.0) to obtain gene expression matrices. Genes with fewer than 200 expressed counts, more than 7,500 genes, or over 20% mitochondrial content were excluded. To integrate cells from different samples, the filtered UMI count matrix was normalized and subjected to principal component analysis (PCA) using SCTransform from the Seurat R package (v4.3.0). The top 20 principal components (PCs) were used for cell clustering *via* the FindNeighbors() and FindClusters() functions. Dimensionality reduction was performed with the RunTSNE and RunUMAP functions, and t-distributed stochastic neighbour embedding (tSNE) or uniform manifold approximation and projection (UMAP) were employed for visualization.

### Cluster annotation and cell scoring

Marker genes for each cluster were identified using the FindAllMarkers() function in the Seurat package. Cell types for each cluster were annotated by comparing the identified markers with known cell type-expressed genes and a database of established marker genes. A total of 13 cell types were identified, including ductal cells, acinar cells, fibroblasts cells, endothelial cells, endocrine cells, hepatocytes, T cells, B cells, plasma cells, macrophages, mast cells, cholangiocytes, and intestinal epithelial cells. Ductal cells, T cells, fibroblasts cells, B cells and macrophages were subsequently extracted for further clustering, dimensionality reduction, and subpopulation analysis. Subpopulations were annotated using the AddModuleScore() function, based on epithelial-mesenchymal transition (EMT), M1, M2, and T cell markers, with annotation genes sourced from the “HALLMARK_EPITHELIAL_MESENCHYMAL_TRANSITION”, “CLASSICAL_M1_VS_ALTERNATIVE_M2_MACROPHAGE_UP”, “CLASSICAL_M1_VS_ALTERNATIVE_M2_MACROPHAGE_DN” in the GSEA database (http://www.gsea-msigdb.org/gsea/msigdb/), ICD marker genes, and a known T cell marker gene database. Gene enrichment analysis of the cluster markers was conducted using clusterProfiler (version 4.6.2) 52, Metascape 53 and GSVA 54.

### Inference of cell fate by pseudotime trajectory analysis

Single-cell trajectory analysis of ductal cells was performed using the monocle package (version 2.26.0). Genes were ranked by differential expression using the differentialGeneTest() function (q-value < 0.01). Dimensionality reduction was performed using the RTree method in the reduceDimension() function, and cells were ordered in pseudotime using the orderCells() function. Results were visualized with the plot_cell() function. Evaluate the differentiation scores of different single-cell subpopulations using cytoTRACE and plot them on the pseudotime trajectory. Branch expression analysis at the branching point was carried out using the BEAM() function, and branched heatmaps were generated with the plot_genes_branchedmap() function. Gene enrichment analysis was conducted using Metascape to uncover significantly altered gene functions over pseudotime.

### Ligand-receptor expression and cell-cell communication analysis

Potential cell-cell communications between various subpopulations were analyzed using the CellChat R package (v1.6.0). A cell object was created from normalized counts data with default parameters using the createCellChat() function. Communication networks were explored by preprocessing data with the identifyOverExpressedGenes(), identifyOverExpressedInteractions(), and projectData() functions. Ligand-receptor interactions and associated signalling pathways were computed using the computeCommunProb() and computeProbPathway() functions. Visualizations of the communication network were generated using netVisual_circle(), netVisual_aggregate(), and netAnalysis_signallingRole_heatmap().

### Data processing of spatial transcriptome sequencing

The 10× libraries were mapped to the GRCh38 human reference genome using the Space Ranger software (v1.20) to obtain the gene expression matrix. Normalization was performed using the NormalizeData() function in Seurat (version 4.3.0), and 2000 highly variable genes were identified using the FindVariableFeatures() function, which were then used as input for batch correction. To remove batch effects, the IntegrateData() function was employed to integrate spots from different samples *via* canonical correlation analysis (CCA). PCA was conducted using the RunPCA() function. The FindNeighbors() and FindClusters() functions in Seurat were applied to cluster spots based on the first 10 principal components, with a resolution of 0.6. Dimensionality reduction and visualization were carried out using the RunTSNE and RunUMAP functions.

### Identification of malignant regions

For further analysis of tumor cell distribution and the gene expression atlas in the primary tumor, deconvolution was performed using the SpaCET.deconvolution() function in the SpaCET package to identify malignant regions in each tissue. Spots were categorized as "stroma", "tumor", and "interface". Expression scores were assigned to spots based on EMT and metastatic dissemination cell-related genes, using the AddModuleScore() function in Seurat with annotated genes from the GSEA database (http://www.gsea-msigdb.org/gsea/msigdb/), particularly the "HALLMARK_EPITHELIAL_MESENCHYMAL_TRANSITION" gene set and metastatic dissemination cell signature genes from the snRNA-seq data. Gene enrichment analysis of signature genes from the interface spots was conducted using clusterProfiler (version 4.6.2). SPOTLight R package (version 0.1.7) was applied to deconvolute spatial transcriptomics data based on snRNA-seq data.

### Cell type enrichment/depletion by multimodal intersection analysis (MIA)

To explore the enrichment of metastatic dissemination cells and immune cells, snRNA-seq and spatial transcriptomics data were integrated using MIA based on the hypergeometric test. Gene sets from snRNA-seq data representing metastatic dissemination cells and immune cells were compared with gene sets from the spatial transcriptomics data representing "stroma", "tumor", and "interface" regions. The overlap between snRNA-seq cell type-specific gene sets and spatial transcriptomics region-specific gene sets was calculated. A lower p-value indicates higher enrichment or depletion of metastatic dissemination cells and immune cells in the spatial transcriptomics regions.

### Data preparation

The clinical features and transcriptomic data for patients with pancreatic adenocarcinoma were obtained from The Cancer Genome Atlas (TCGA) database (https://portal.g.cancer.gov/), which includes 4 normal tissue samples and 179 pancreatic adenocarcinoma samples. For normalization, the gene expression levels in the RNAseq data were transformed into TPM (Transcripts Per Million) format using STAR 55. The TPM-formatted RNAseq data of TCGA and GTEx normalized by the Toil pipeline 56 can be downloaded from UCSC XENA (https://xenabrowser.net/datapages/).

### Construction of the prognostic model

Univariate Cox regression analysis was performed on the metastatic dissemination cell-related expressed genes, and variables with a p-value < 0.05 were included for feature gene screening related to OS. The Least Absolute Shrinkage and Selection Operator (LASSO) regression model was applied to reduce the number of genes in the final model. Candidate genes were selected based on 10-fold cross-validation. The risk factor plot and survival curve for patients were generated using the ggrisk and survival packages in R software. The receiver operating characteristic (ROC) curve was plotted using the pROC package to evaluate the risk score's performance in predicting 1-, 3-, and 5-year OS of patients with pancreatic adenocarcinoma, and visualized using the ggplot2 package. A calibration curve for 1, 2, and 3 years was plotted using the rms package to assess the predictive accuracy of the prognostic model. The decision curve analysis (DCA) for the model was calculated using the stdca.R package to evaluate its clinical applicability. Clinical information, including staging and gene expression, was integrated using the rms package to construct a nomogram for comprehensive assessment of the 1-year, 3-year, and 5-year survival probability of patients with pancreatic adenocarcinoma. A ROC curve was plotted to evaluate the prognostic predictive performance of the nomogram.

### Evaluation of the immune microenvironment

The ssGSEA, xCell, and CIBERSORT algorithm was utilized to assess the infiltration status of immune cells in each TCGA-PAAD sample. Samples were classified into high-risk and low-risk groups based on the CITED4 expression or risk scores derived from LASSO regression analysis, and the relative content of immune cells between these two groups was compared.

### Evaluation of subtype clinical characteristics

To identify independent prognostic factors for the prognostic genes, the variables were further evaluated using multivariate Cox regression analysis. The TCGA-PAAD samples were stratified by clinicopathological characteristics such as age (> 65 and ≤ 65 years) and T stage, and then divided into high and low expression groups for CITED4 or CIRBP. Kaplan-Meier survival analysis was conducted on the high and low expression groups of clinical features to further evaluate the independent prognostic performance of CITED4 or CIRBP in each subgroup.

### Prediction of target genes regulated by CITED4

The target genes regulated by the EP300 and TFAP2 transcription factors associated with the CITED4 gene were predicted using the Transcription Regulatory Relationships Unraveled by Sentence-based Text mining (TRRUST) database. KEGG functional annotation of the target genes was performed using Metascape to explore their biological significance.

### Immunohistochemical staining

FFPE PDAC tissues were used for immunohistochemical staining. Tissue sections (4 μm thick) were baked onto slides at 65 °C overnight. After deparaffinization and hydration with xylene and graded ethanol, antigen retrieval was performed by boiling in citrate buffer for 15 min, followed by endogenous peroxidase blocking using 3% hydrogen peroxide. The slides were then blocked with 5% goat serum for 1 h to prevent nonspecific antibody binding, followed by overnight incubation at 4 ℃ with anti-MIF primary antibody (Santa Cruz, D-2, 1:200) or anti-CITED4 primary antibody (Novus Biologicals, NB110-41572, 1:500). After washing with TBST, the slides were incubated with horseradish peroxidase (HRP)-conjugated goat anti-rabbit/mouse secondary antibodies for 1 h at room temperature. The slides were then stained with DAB and hematoxylin, counterstained with nuclei, dehydrated, and scanned using a 3Dhistech Pannoramic Scan system. The MIF or CITED4 expression score in FFPE samples was calculated by multiplying the total score of the percentage of MIF or CITED4-positive staining (0-5% = 0, 6-25% = 1, 26-50% = 2, 51-75% = 3, >75% = 4) and the staining intensity, graded into four levels: no staining = 0, weak = 1, moderate = 2, strong = 3.

### Multiplexed immunofluorescence staining

Multiplexed immunofluorescence staining was performed using a 7-color immunohistochemistry kit (PhenoVision Bio Co., Ltd) according to the manufacturer's instructions. PDAC and matched liver metastasis tissue sections were deparaffinized with xylene, hydrated with graded ethanol, and antigen retrieval was performed in citric acid buffer (0.01 M, pH 6.0). Endogenous peroxidase was blocked with hydrogen peroxide, followed by sealing with blocking agents. The sections were incubated with primary antibodies (CD4, CD8, FOXP3, CD20, panCK, and VIM from ZSbio or CST) for 30 min at room temperature, followed by a 10 min incubation with PVB tyramide signal amplification fluorophore (PVB 480, PVB 520, PVB 50, PVB 620, PVB 690, and PVB 780). The slides were counterstained with 4′,6-diamidino-2-phenylindole (PhenoVision Bio Co., Ltd). Scanning and analysis were performed using the PhenoImage system (Akoya Biosciences).

### Cell culture

The L3.6p1 cell line was obtained from American Type Culture Collection (ATCC) and authenticated by short tandem repeat (STR) profiling. Cells were cultured in Dulbecco's modified Eagle's medium (DMEM, Gibco, Life Technologies, Waltham, MA, USA) supplemented with 10% fetal bovine serum (FBS, Gibco, Life Technologies, Waltham, MA, USA) and 1% penicillin-streptomycin mixture (P/S, Thermo Fisher Scientific, Waltham, MA, USA). Cells were maintained at 37 °C in a humidified atmosphere with 5% CO₂.

### siRNA knockdown

Cells were seeded in 6-well plates and grown to 70% confluence. siRNA duplexes (50 nM) targeting CITED4 and negative control siRNA were transfected using Lipofectamine RNAiMAX (Invitrogen) according to the manufacturer's protocol. Briefly, siRNA and transfection reagent were diluted in Opti-MEM, incubated for 5 min, and combined before adding to cells. After 72 h, transfection efficiency was verified by western blot. The target sequences specific for CITED4 were used: UGGGCCAGAGCGAGUUCGACU.

### Transwell assays

Transwell assays evaluated cell migration using polycarbonate membranes with 8 μm pores. Cells were seeded in serum-free medium in the upper chamber, with the lower chamber containing 10% FBS as chemoattractant. After incubation at 37 °C for 48 h, non-migrated cells were removed, and membranes were fixed with 4% paraformaldehyde and stained with 0.1% crystal violet. Migrated cells were counted in five random fields per membrane.

### Scratch wound healing assays

Cells were grown to 90% confluence, and a scratch was made using a sterile pipette tip. The plate was washed to remove debris, and fresh medium was added. Wound closure was monitored at 24 h. Images were captured at predefined locations, and the wound area was quantified using ImageJ. The percentage of wound closure was calculated relative to the initial wound area.

### Statistical analysis

Bioinformatics and statistical analysis were performed using R language (version 4.2.1) (https://www.r-project.org/), SPSS (version 23.0), and GraphPad Prism (version 8.0) software. Statistical tests included the t-test and Chi-square test. Results were considered statistically significant as follows: *p < 0.05, **p < 0.01, ***p < 0.001, ****p < 0.0001.

## Supplementary Material

Supplementary figures.

Supplementary tables.

## Figures and Tables

**Table 1:** Correlation between CITED4 protein expression and clinical characteristics of patients with pancreatic adenocarcinoma.

Indicators	Low expression of CITED4	High expression of CITED4	*P* value
Gender (n)			0.512
Male	35	8	
Female	27	4	
Age (n)			0.466
<65	24	6	
≥65	38	6	
Differentiation (n)			0.763
Low	7	1	
High/middle	55	11	
T stage (n)			0.487
T1-2	10	1	
T3-4	52	11	
N stage (n)			0.512
N0	35	8	
N1-3	27	4	
M stage (n)			<0.0001
M0	59	7	
M1	3	5	
Clinicopathologic stage (n)			<0.0001
Stage I/II	59	7	
Stage III/IV	3	5	

## Abbreviations

PT: Primary tumor
LM: Liver metastasis
IHC: Immunohistochemistry
EMT: Epithelial-mesenchymal transition
PDAC: Pancreatic ductal adenocarcinoma
scRNA seq: Single-cell RNA sequencing
TME: tumor microenvironment
snRNA-seq: Single-nucleus RNA sequencing
ST: Spatial transcriptomics
Tregs: Regulatory T cells
FFPE: Formalin-fixed, paraffin-embedded
CNV: Copy number variation
TIL: tumor infiltrating lymphocyte
PMDS: Pancreatic metastatic dissemination signature
ECM: Extracellular matrix
OS: Overall survival
PAAD: Pancreatic adenocarcinoma
MDRGs: Metastatic dissemination-related genes
DSS: Disease specific survival
CITED: CBP/p300-interacting transactivator with glutamic acid and aspartic acid-rich tail
TLS: tertiary lymphoid structure

## References

[1] Beatty GL, Werba G, Lyssiotis CA, Simeone DM (2021). The biological underpinnings of therapeutic resistance in pancreatic cancer. Genes Dev.

[2] Kleeff J, Korc M, Apte M, La Vecchia C, Johnson CD, Biankin AV (2016). Pancreatic cancer. Nat Rev Dis Primers.

[3] Houg DS, Bijlsma MF (2018). The hepatic pre-metastatic niche in pancreatic ductal adenocarcinoma. Mol Cancer.

[4] Chandana S, Babiker HM, Mahadevan D (2019). Therapeutic trends in pancreatic ductal adenocarcinoma (PDAC). Expert Opin Investig Drugs.

[5] Bellomo G, Rainer C, Quaranta V, Astuti Y, Raymant M, Boyd E (2022). Chemotherapy-induced infiltration of neutrophils promotes pancreatic cancer metastasis via Gas6/AXL signalling axis. Gut.

[6] Sherman MH, Beatty GL (2023). Tumor Microenvironment in Pancreatic Cancer Pathogenesis and Therapeutic Resistance. Annu Rev Pathol.

[7] Xie Z, Gao Y, Ho C, Li L, Jin C, Wang X (2022). Exosome-delivered CD44v6/C1QBP complex drives pancreatic cancer liver metastasis by promoting fibrotic liver microenvironment. Gut.

[8] Chijimatsu R, Kobayashi S, Takeda Y, Kitakaze M, Tatekawa S, Arao Y (2022). Establishment of a reference single-cell RNA sequencing dataset for human pancreatic adenocarcinoma. iScience.

[9] Li J, Wei T, Ma K, Zhang J, Lu J, Zhao J (2024). Single-cell RNA sequencing highlights epithelial and microenvironmental heterogeneity in malignant progression of pancreatic ductal adenocarcinoma. Cancer Lett.

[10] Azevedo-Pouly AC, Elgamal OA, Schmittgen TD (2014). RNA isolation from mouse pancreas: a ribonuclease-rich tissue. J Vis Exp.

[11] Migliorini A, Ge S, Atkins MH, Oakie A, Sambathkumar R, Kent G (2024). Embryonic macrophages support endocrine commitment during human pancreatic differentiation. Cell Stem Cell.

[12] Slyper M, Porter CBM, Ashenberg O, Waldman J, Drokhlyansky E, Wakiro I (2020). A single-cell and single-nucleus RNA-Seq toolbox for fresh and frozen human tumors. Nat Med.

[13] Yousuf S, Qiu M, Voith von Voithenberg L, Hulkkonen J, Macinkovic I, Schulz AR (2023). Spatially Resolved Multi-Omics Single-Cell Analyses Inform Mechanisms of Immune Dysfunction in Pancreatic Cancer. Gastroenterology.

[14] Hwang WL, Jagadeesh KA, Guo JA, Hoffman HI, Yadollahpour P, Reeves JW (2022). Single-nucleus and spatial transcriptome profiling of pancreatic cancer identifies multicellular dynamics associated with neoadjuvant treatment. Nat Genet.

[15] Luo W, Wen T, Qu X (2024). Tumor immune microenvironment-based therapies in pancreatic ductal adenocarcinoma: time to update the concept. J Exp Clin Cancer Res.

[16] Steele NG, Carpenter ES, Kemp SB, Sirihorachai VR, The S, Delrosario L (2020). Multimodal Mapping of the Tumor and Peripheral Blood Immune Landscape in Human Pancreatic Cancer. Nat Cancer.

[17] Liudahl SM, Betts CB, Sivagnanam S, Morales-Oyarvide V, da Silva A, Yuan C (2021). Leukocyte Heterogeneity in Pancreatic Ductal Adenocarcinoma: Phenotypic and Spatial Features Associated with Clinical Outcome. Cancer Discov.

[18] Piper M, Van Court B, Mueller A, Watanabe S, Bickett T, Bhatia S (2022). Targeting Treg-Expressed STAT3 Enhances NK-Mediated Surveillance of Metastasis and Improves Therapeutic Response in Pancreatic Adenocarcinoma. Clin Cancer Res.

[19] Park JK, Jeong HO, Kim H, Choi JH, Lee EM, Kim S (2024). Single-cell transcriptome analysis reveals subtype-specific clonal evolution and microenvironmental changes in liver metastasis of pancreatic adenocarcinoma and their clinical implications. Mol Cancer.

[20] Zhu H, Xu J, Wang W, Zhang B, Liu J, Liang C (2024). Intratumoral CD38(+)CD19(+)B cells associate with poor clinical outcomes and immunosuppression in patients with pancreatic ductal adenocarcinoma. EBioMedicine.

[21] Takahashi R, Macchini M, Sunagawa M, Jiang Z, Tanaka T, Valenti G (2021). Interleukin-1beta-induced pancreatitis promotes pancreatic ductal adenocarcinoma via B lymphocyte-mediated immune suppression. Gut.

[22] Peng J, Sun BF, Chen CY, Zhou JY, Chen YS, Chen H (2019). Single-cell RNA-seq highlights intra-tumoral heterogeneity and malignant progression in pancreatic ductal adenocarcinoma. Cell Res.

[23] Zhang S, Fang W, Zhou S, Zhu D, Chen R, Gao X (2023). Single cell transcriptomic analyses implicate an immunosuppressive tumor microenvironment in pancreatic cancer liver metastasis. Nat Commun.

[24] Sun Y, Wu L, Zhong Y, Zhou K, Hou Y, Wang Z (2021). Single-cell landscape of the ecosystem in early-relapse hepatocellular carcinoma. Cell.

[25] MacParland SA, Liu JC, Ma XZ, Innes BT, Bartczak AM, Gage BK (2018). Single cell RNA sequencing of human liver reveals distinct intrahepatic macrophage populations. Nat Commun.

[26] Ingersoll SA, Ayyadurai S, Charania MA, Laroui H, Yan Y, Merlin D (2012). The role and pathophysiological relevance of membrane transporter PepT1 in intestinal inflammation and inflammatory bowel disease. Am J Physiol Gastrointest Liver Physiol.

[27] Xie Z, Niu L, Zheng G, Du K, Dai S, Li R (2023). Single-cell analysis unveils activation of mast cells in colorectal cancer microenvironment. Cell Biosci.

[28] Ji L, Guo W (2023). Single-cell RNA sequencing highlights the roles of C1QB and NKG7 in the pancreatic islet immune microenvironment in type 1 diabetes mellitus. Pharmacol Res.

[29] Onieva JL, Xiao Q, Berciano-Guerrero MA, Laborda-Illanes A, de Andrea C, Chaves P (2022). High IGKC-Expressing Intratumoral Plasma Cells Predict Response to Immune Checkpoint Blockade. Int J Mol Sci.

[30] Yang F, Ma J, Zhu D, Wang Z, Li Y, He X (2023). The Role of S100A6 in Human Diseases: Molecular Mechanisms and Therapeutic Potential. Biomolecules.

[31] Gulati GS, Sikandar SS, Wesche DJ, Manjunath A, Bharadwaj A, Berger MJ (2020). Single-cell transcriptional diversity is a hallmark of developmental potential. Science.

[32] Trapnell C, Cacchiarelli D, Grimsby J, Pokharel P, Li S, Morse M (2014). The dynamics and regulators of cell fate decisions are revealed by pseudotemporal ordering of single cells. Nat Biotechnol.

[33] Jin S, Guerrero-Juarez CF, Zhang L, Chang I, Ramos R, Kuan CH (2021). Inference and analysis of cell-cell communication using CellChat. Nat Commun.

[34] Ru B, Huang J, Zhang Y, Aldape K, Jiang P (2023). Estimation of cell lineages in tumors from spatial transcriptomics data. Nat Commun.

[35] Elosua-Bayes M, Nieto P, Mereu E, Gut I, Heyn H (2021). SPOTlight: seeded NMF regression to deconvolute spatial transcriptomics spots with single-cell transcriptomes. Nucleic Acids Res.

[36] Moncada R, Barkley D, Wagner F, Chiodin M, Devlin JC, Baron M (2020). Integrating microarray-based spatial transcriptomics and single-cell RNA-seq reveals tissue architecture in pancreatic ductal adenocarcinomas. Nature Biotechnology.

[37] Ma RY, Black A, Qian BZ (2022). Macrophage diversity in cancer revisited in the era of single-cell omics. Trends Immunol.

[38] Park SY, Kim IS (2019). Harnessing immune checkpoints in myeloid lineage cells for cancer immunotherapy. Cancer Lett.

[39] Biffi G, Tuveson DA (2021). Diversity and Biology of Cancer-Associated Fibroblasts. Physiol Rev.

[40] Cords L, Tietscher S, Anzeneder T, Langwieder C, Rees M, de Souza N (2023). Cancer-associated fibroblast classification in single-cell and spatial proteomics data. Nat Commun.

[41] Yang Y, Chen X, Pan J, Ning H, Zhang Y, Bo Y (2024). Pan-cancer single-cell dissection reveals phenotypically distinct B cell subtypes. Cell.

[42] Amisaki M, Zebboudj A, Yano H, Zhang SL, Payne G, Chandra AK (2025). IL-33-activated ILC2s induce tertiary lymphoid structures in pancreatic cancer. Nature.

[43] Zhang L, Wang Y, Sha Y, Zhang B, Zhang R, Zhang H (2021). CITED4 enhances the metastatic potential of lung adenocarcinoma. Thorac Cancer.

[44] Han H, Cho J-W, Lee S, Yun A, Kim H, Bae D (2018). TRRUST v2: an expanded reference database of human and mouse transcriptional regulatory interactions. Nucleic Acids Research.

[45] Li Z, Lai X, Fu S, Ren L, Cai H, Zhang H (2022). Immunogenic Cell Death Activates the Tumor Immune Microenvironment to Boost the Immunotherapy Efficiency. Adv Sci (Weinh).

[46] Borchers A, Pieler T (2010). Programming pluripotent precursor cells derived from Xenopus embryos to generate specific tissues and organs. Genes (Basel).

[47] Dasgupta A, Lim AR, Ghajar CM (2017). Circulating and disseminated tumor cells: harbingers or initiators of metastasis?. Mol Oncol.

[48] Mohme M, Riethdorf S, Pantel K (2017). Circulating and disseminated tumour cells - mechanisms of immune surveillance and escape. Nat Rev Clin Oncol.

[49] de Visser KE, Joyce JA (2023). The evolving tumor microenvironment: From cancer initiation to metastatic outgrowth. Cancer Cell.

[50] Ullman NA, Burchard PR, Dunne RF, Linehan DC (2022). Immunologic Strategies in Pancreatic Cancer: Making Cold Tumors Hot. J Clin Oncol.

[51] Xu Y, Fu J, Henderson M, Lee F, Jurcak N, Henn A (2023). CLDN18.2 BiTE Engages Effector and Regulatory T Cells for Antitumor Immune Response in Preclinical Models of Pancreatic Cancer. Gastroenterology.

[52] Yu G, Wang LG, Han Y, He QY (2012). clusterProfiler: an R package for comparing biological themes among gene clusters. OMICS.

[53] Zhou Y, Zhou B, Pache L, Chang M, Khodabakhshi AH, Tanaseichuk O (2019). Metascape provides a biologist-oriented resource for the analysis of systems-level datasets. Nat Commun.

[54] Hanzelmann S, Castelo R, Guinney J (2013). GSVA: gene set variation analysis for microarray and RNA-seq data. BMC Bioinformatics.

[55] Dobin A, Davis CA, Schlesinger F, Drenkow J, Zaleski C, Jha S (2013). STAR: ultrafast universal RNA-seq aligner. Bioinformatics.

[56] Vivian J, Rao AA, Nothaft FA, Ketchum C, Armstrong J, Novak A (2017). Toil enables reproducible, open source, big biomedical data analyses. Nat Biotechnol.

