# Supplementary material for: Single-nucleus RNA sequencing and spatial transcriptomics reveal an immunosuppressive tumor microenvironment related to metastatic dissemination during pancreatic cancer liver metastasis
Source: Theranostics. 2025 Apr 13;15(11):5337–57. doi: 10.7150/thno.108925 (PMC12036881; doi:10.7150/thno.108925)
Supplement: Supplementary file 1 — Supplementary figures. [file thnov15p5337s1.pdf]

# Supplementary Figures and Figure Legends

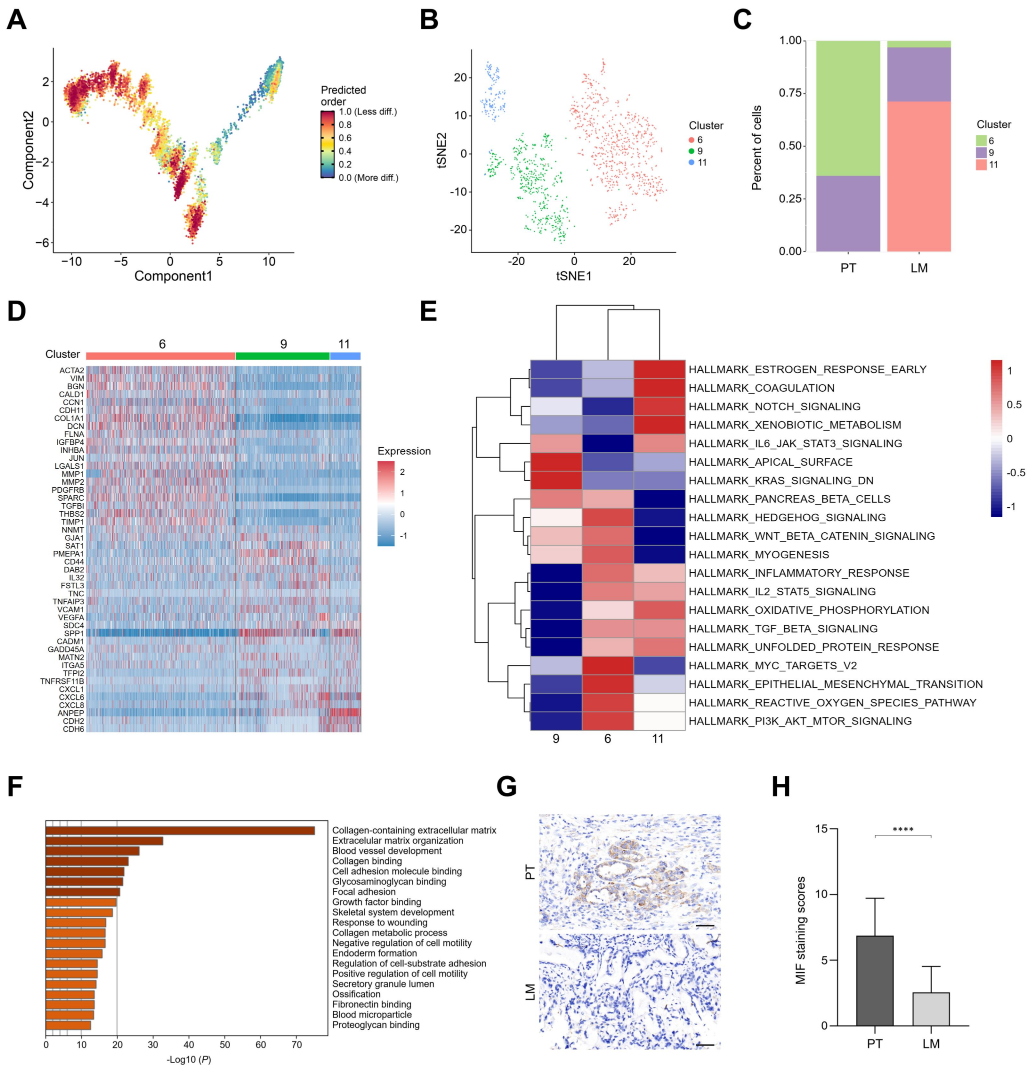

**Figure S1. Characterization of ductal cells related to Figure 2. (A)** The cell differentiation dynamics of ductal cell subpopulations during evolutionary trajectories. **(B)** UMAP plot of subpopulations 6, 9, and 11 derived from the the ductal cells clusters in Figure 2A. **(C)** Bar plots showing the cell proportion of subpopulations 6, 9, and 11 from PT and LM. **(D)** Heatmap showing the expression levels of metastasis-related genes in subpopulations 6, 9, and 11. **(E)** GSVA enrichment analysis of biological functions and relevant signaling pathways in subpopulations 6, 9, and 11 based on Hallmark gene sets. **(F)** GO enrichment analysis of differentially expressed genes contrasting subpopulation 6 with subpopulations 9 and 11. **(G)** Immunohistochemistry detection of MIF expression in pancreatic adenocarcinoma primary tumors and liver metastatic lesions. Scale bars, 50 μm. **(H)** Quantified staining scores of MIF in **(G)**, with statistical testing by t-test (****, $p < 0.0001$).

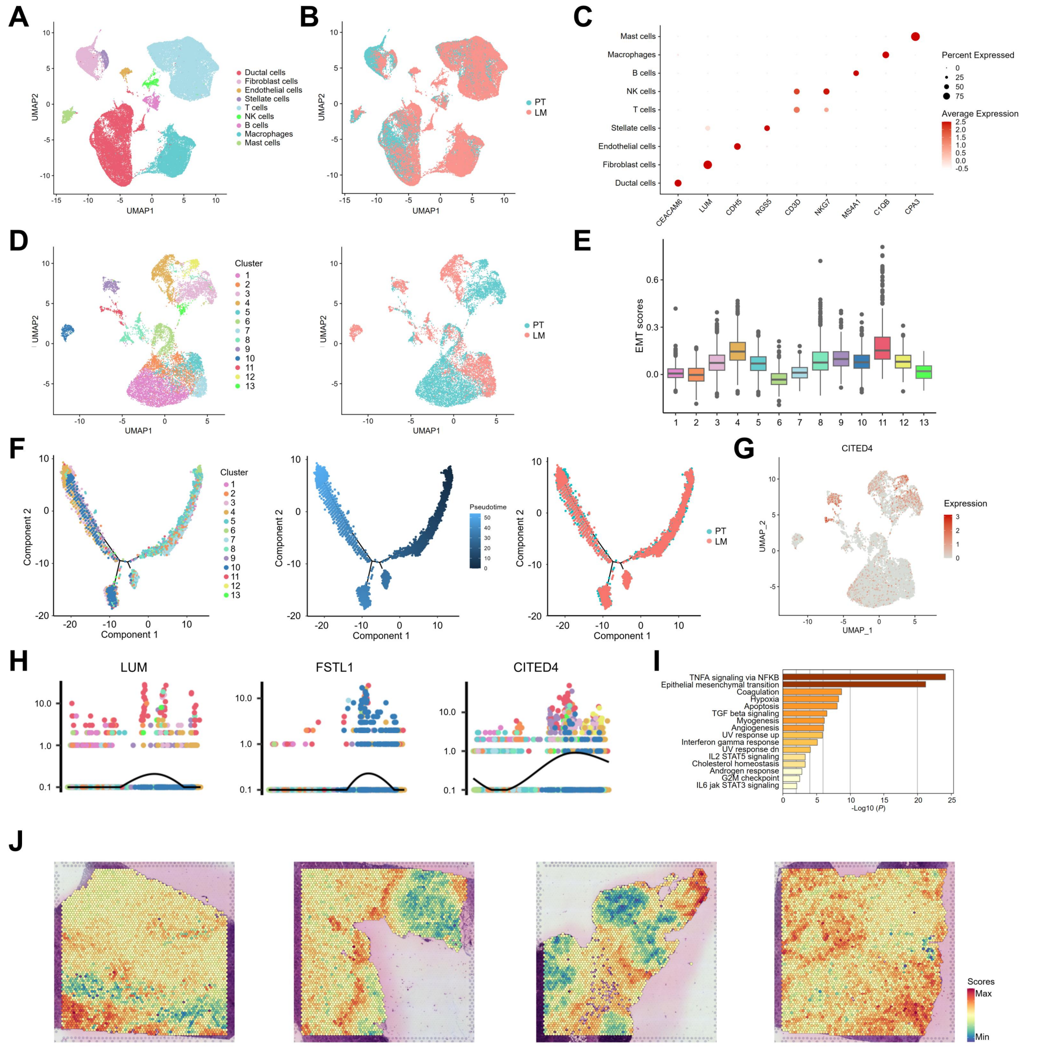


**Figure S2. Single-cell transcriptomic landscape of PDAC and transcriptional signatures of ductal cell subpopulations. (A)** UMAP plots of high-quality cells from primary tumor (PT) and liver metastatic tumors (LM), showing cell types, colour-coded by inferred cell types. **(B)** UMAP plots of the cell atlas showing their sample origins, colour-coded according to their tissues. **(C)** Dotplot showing the expression levels of major differentially expressed genes in the 9 cell types. **(D)** UMAP plot of the ductal cells landscape. Cells were coloured according to their clusters (left) or tissues (right). **(E)** Box plots showing EMT scores for each ductal cell subcluster. **(F)** Monocle pseudotime trajectory analysis of ductal cells clusters during the progression process. **(G)** Feature plots showing the normalized expression of CITED4 in ductal cells. **(H)** Dot plots of LUM, FSTL1 and CITED4 along evolutionary trajectories. **(I)** Hallmark pathway enrichment analysis of differentially expressed genes between subpopulation 11 and other ductal cell subclusters. **(J)** Spatial feature plots of subpopulation 11 in PDAC spatial transcriptomic samples.

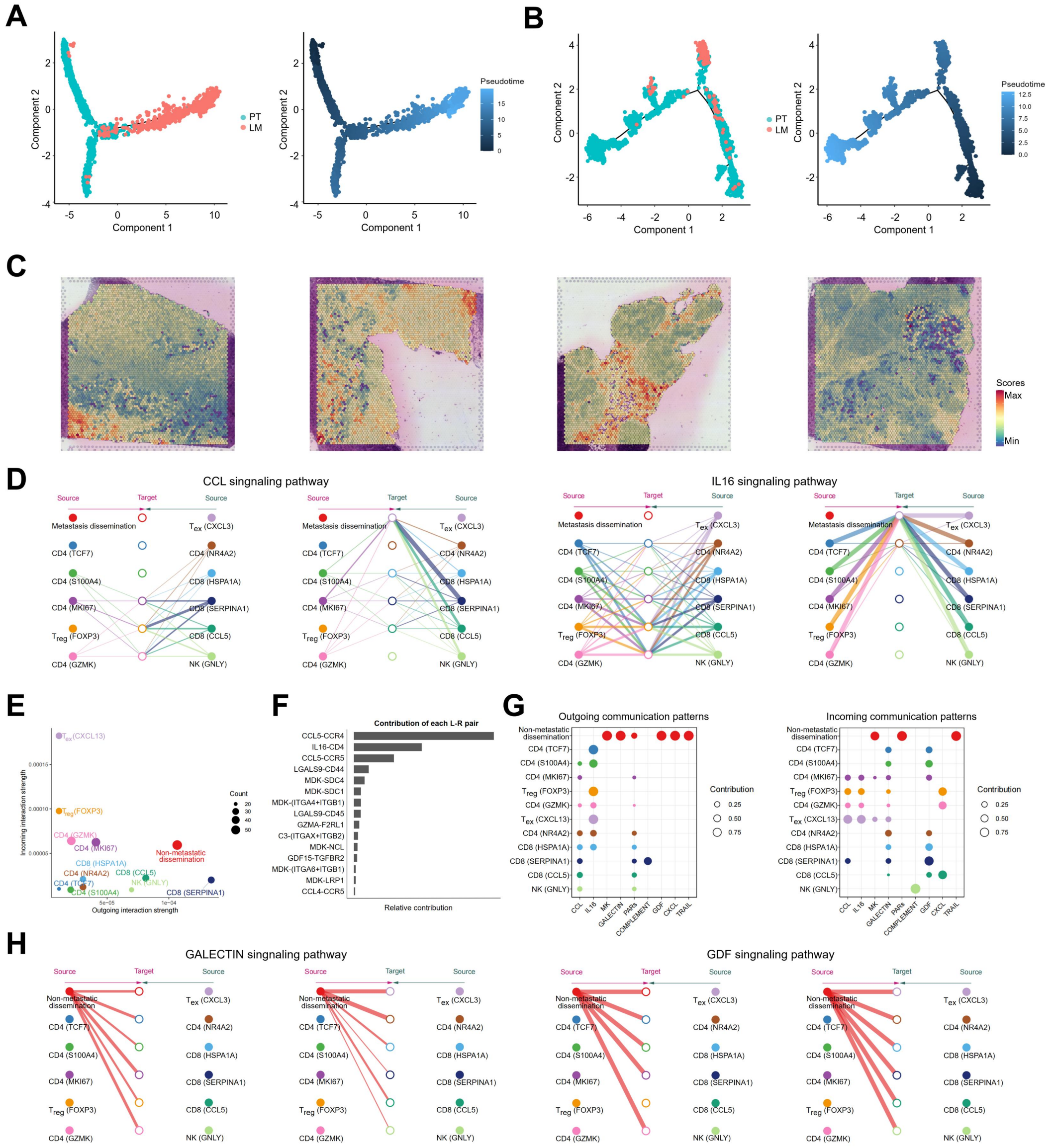

**Figure S3. Monocle pseudotime trajectory analysis of T cells, spatial features and intercellular ligand–receptor prediction between ductal cells and lymphoid cells. (A)** Monocle pseudotime trajectory analysis of CD8 cells related to Figure 3D. **(B)** Monocle pseudotime trajectory analysis of CD4 cells related to Figure 3E. **(C)** SPOTLight-based spatial deconvolution of metastatic dissemination cells in PDAC spatial transcriptomics datasets. **(D)** Hierarchical plot showing the strength of the CCL (left) and IL16 (right) signalling pathway between metastatic dissemination cells and lymphoid cells. **(E)** Scatter plot showing the relative strength of the outgoing and incoming signalling pathways in non-metastatic dissemination cells and lymphoid cells. **(F)** Contribution of each ligand-receptor pair in non-metastatic dissemination cells and lymphoid cells. **(G)** Bubble plots showing the specific ligand-receptor pairs of outgoing and incoming signalling pathways in non-metastatic dissemination cells and lymphoid cells. **(H)** Hierarchical plot showing the strength of the GALECTIN (left) and GDF (right) signalling pathway between non-metastatic dissemination cells and lymphoid cells.

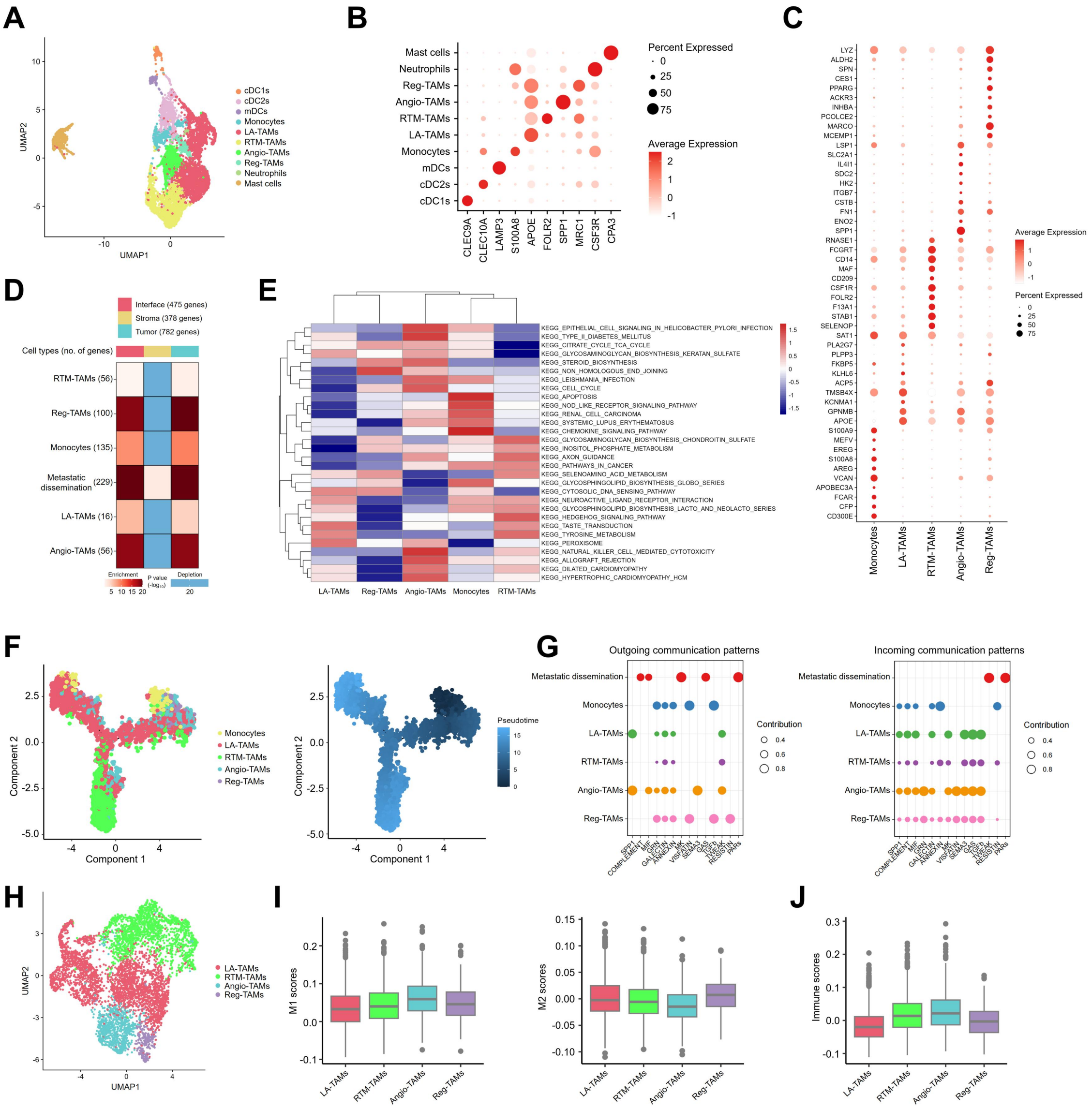


**Figure S4. Transcriptional signatures of myeloid cells identified by snRNA-seq. (A)** UMAP plot of the myeloid cells landscape, coloured by subcluster. **(B)** Dotplot showing the expression levels of selected marker genes in the subclusters. **(C)** Dotplot showing the expression levels of top 10 differentially expressed genes in the TAMs subclusters. **(D)** MIA map of overlap between snRNA-seq-defined metastatic dissemination cells, TAMs, and ST-defined tumor, interface, and stroma regions. **(E)** GSVA enrichment analysis of biological functions and relevant signaling pathways in TAMs based on KEGG gene sets. **(F)** Monocle pseudotime trajectory analysis of TAMs during the progression process. **(G)** Bubble plots showing the specific ligand-receptor pairs of outgoing and incoming signalling pathways in metastatic dissemination cells and TAMs. **(H)** UMAP plot of the TAMs landscape. **(I)** Box plots showing M1 scores (left) and M2 scores (right) for each TAM subcluster. **(J)** Box plots showing immune checkpoint-related gene scores for each TAM subcluster.

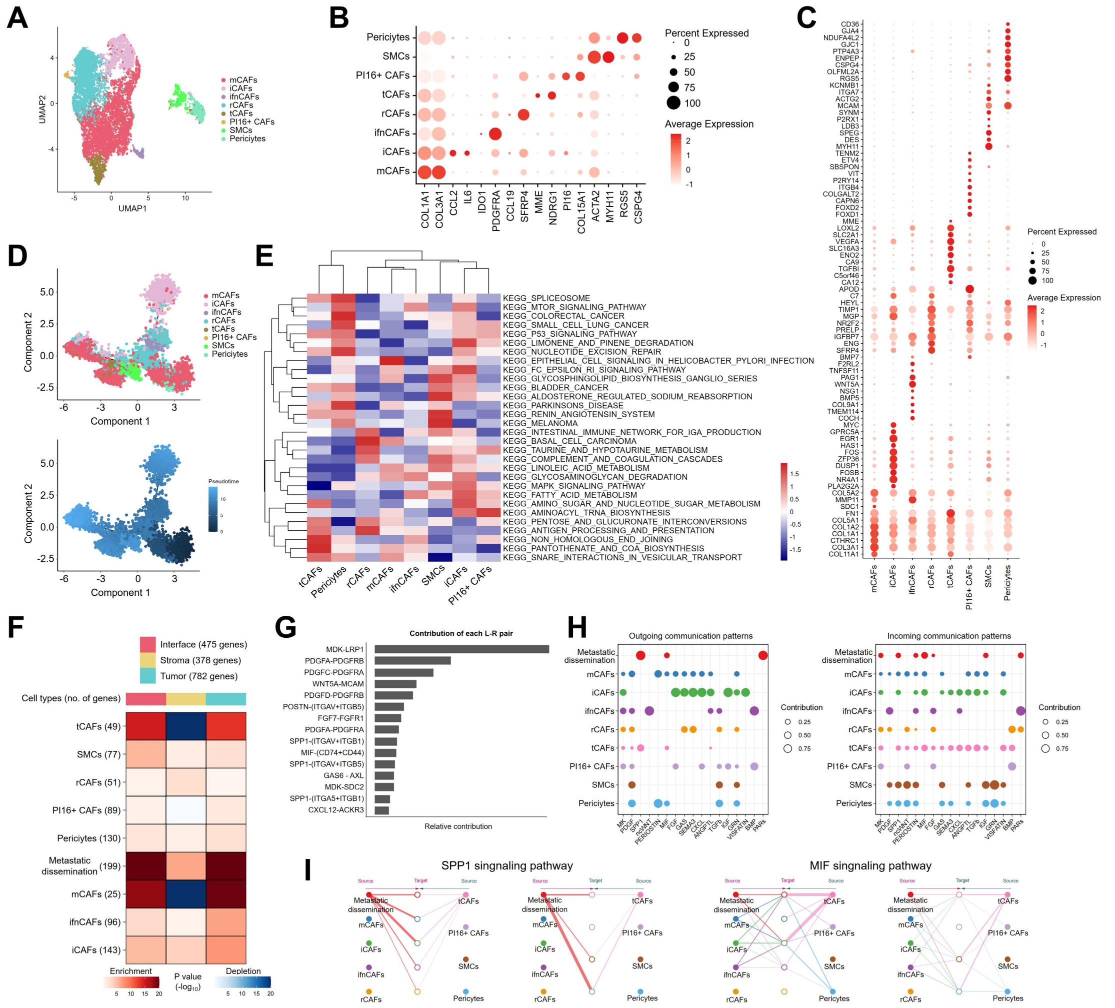

**Figure S5. Transcriptional signatures of fibroblast cells identified by snRNA-seq. (A)** UMAP plot of the fibroblast cells landscape, coloured by subcluster. **(B)** Dotplot showing the expression levels of selected marker genes in the subclusters. **(C)** Dotplot showing the expression levels of top 10 differentially expressed genes in the CAFs subclusters. **(D)** Monocle pseudotime trajectory analysis of CAFs during the progression process. **(E)** GSVA enrichment analysis of biological functions and relevant signaling pathways in CAFs based on KEGG gene sets. **(F)** MIA map of overlap between snRNA-seq-defined metastatic dissemination cells, CAFs, and ST-defined tumor, interface, and stroma regions. **(G)** Contribution of each ligand-receptor pair in metastatic dissemination cells and CAFs. **(H)** Bubble plots showing the specific ligand-receptor pairs of outgoing and incoming signalling pathways in metastatic dissemination cells and CAFs. **(I)** Hierarchical plot showing the strength of the SPP1 (left) and MIF (right) signalling pathway between metastatic dissemination cells and CAFs.

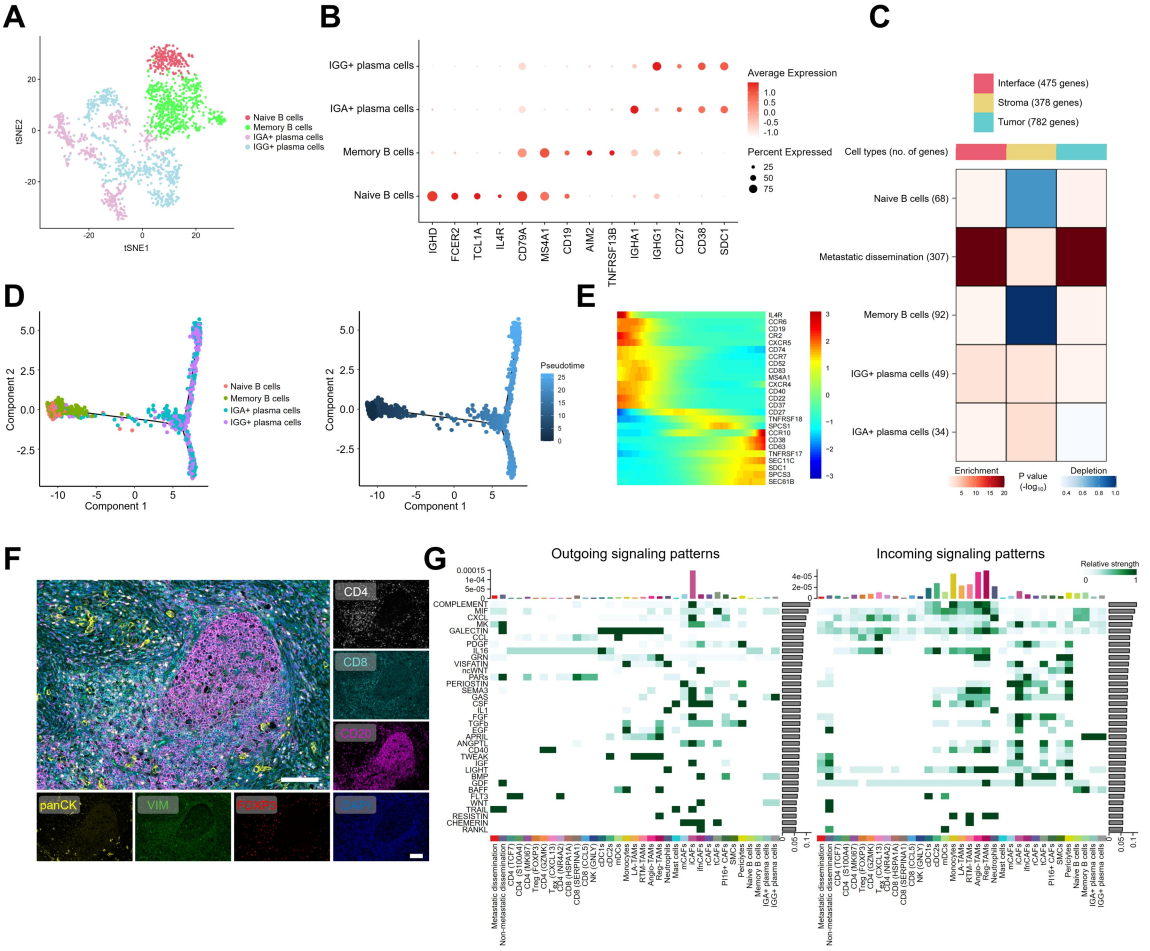


**Figure S6. Transcriptional signatures of B cells identified by snRNA-seq. (A)** UMAP plot of the B cells landscape, coloured by subcluster. **(B)** Dotplot showing the expression levels of selected marker genes in the subclusters. **(C)** MIA map of overlap between snRNA-seq-defined metastatic dissemination cells, B cells, and ST-defined tumor, interface, and stroma regions. **(D)** Monocle pseudotime trajectory analysis of B cells subclusters during the progression process. **(E)** Heatmap of top differentially expressed genes along evolutionary trajectories. **(F)** Multiplex immunofluorescence assay of pancreatic adenocarcinoma tissues stained for CD4, CD8, CD20, FOXP3, VIM, Pan-CK, and DAPI. Scale bars, 100 μm. **(G)** Heatmap showing the relative strength of the outgoing and incoming signalling pathways in each cell subclusters.

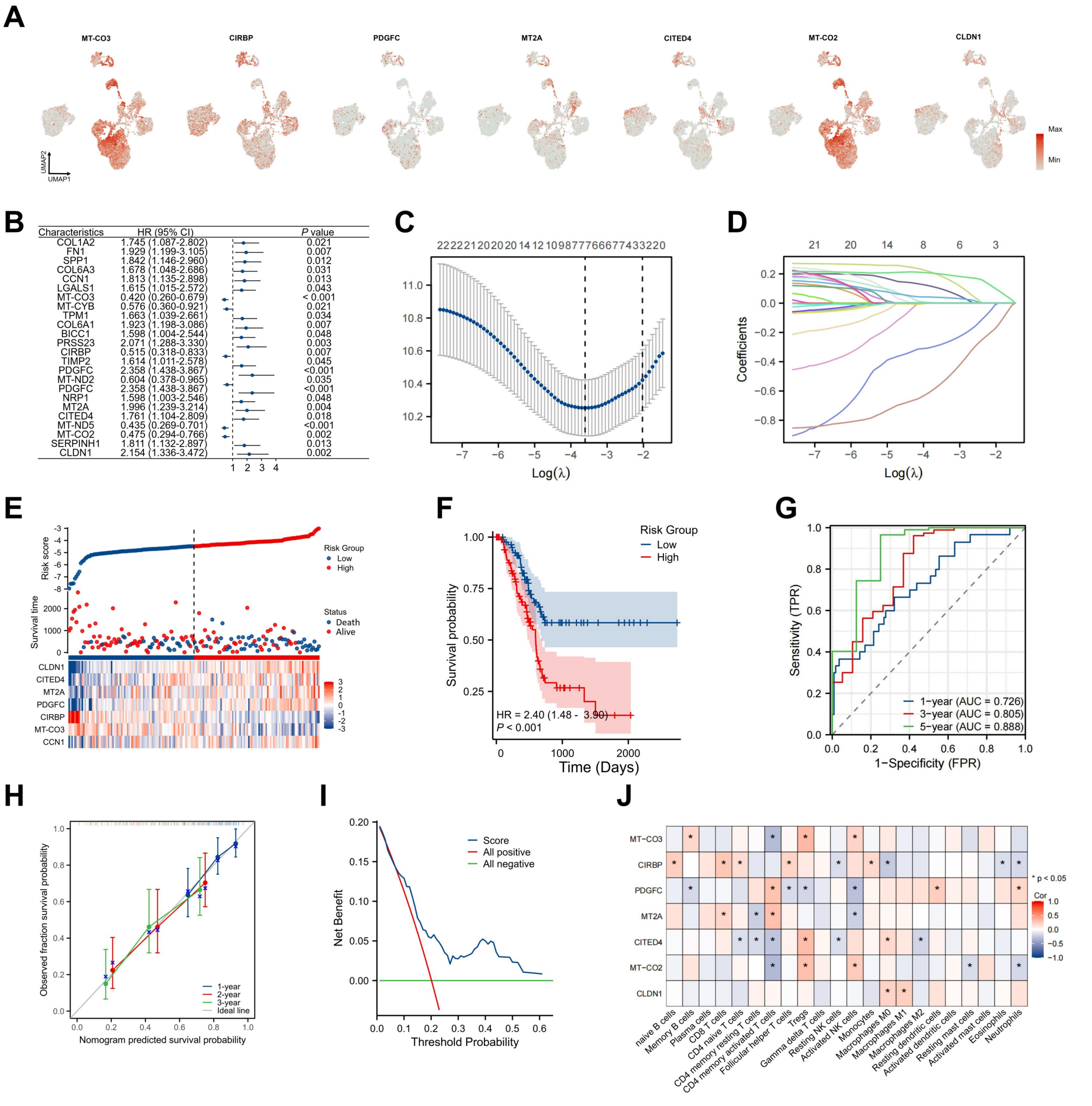


**Figure S7. Development of a metastatic dissemination-related prognostic signature based on disease-specific survival (DSS) analysis. (A)** Feature plots showing the normalized expression of seven key MDRGs in ductal cells related to Figure 2A. **(B)** Univariate Cox regression analysis for MDRGs significantly associated with DSS. **(C, D)** LASSO regression analysis identified DSS-related candidate prognostic genes. **(E)** Risk score, survival status, and heatmap of the expression levels of the 7 candidate prognostic MDRGs in patients with pancreatic adenocarcinoma. **(F)** Kaplan-Meier survival analysis for DSS between the high- and low-risk groups. **(G)** ROC curves of the DSS-prognostic model for predicting 1-, 3-, and 5-year survival rates. **(H)** Calibration curve of the DSS-prognostic model. **(I)** Decision curve analysis (DCA) for the evaluation of the DSS-prognostic model. **(J)** Heatmap visualization of correlation analysis between MDRGs and immune infiltration data based on the CIBERSORT algorithm.

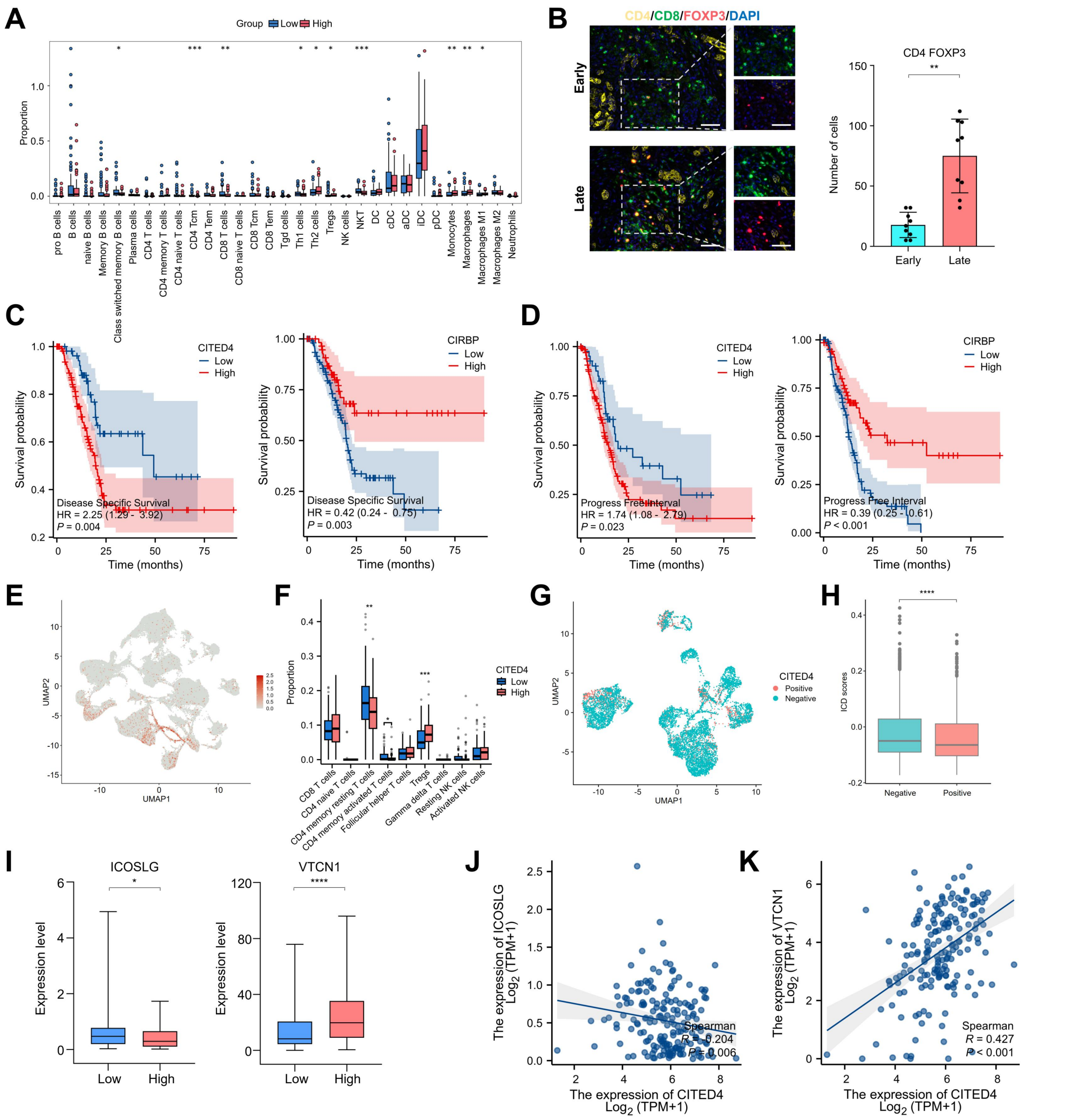


**Figure S8. Development of a prognostic model for characterizing the immune microenvironment landscape in pancreatic adenocarcinoma. (A)** Boxplot showing the xCell scores of immune cell subsets in high- and low-risk groups related to Figure 5L. **(B)** Immunofluorescence assay for PanCK, CD4, FOXP3, and DAPI in PT and LM samples. Scale bars, 50 µm. Statistical analysis of the number of CD4 FOXP3 T cells in PT and LM, statistical testing was performed using the t-test (**, $p < 0.01$). **(C)** Kaplan-Meier curves showing DSS of patients with pancreatic adenocarcinoma according to CITED4 expression (left) or CIRBP expression (right). Grouping via P-value minimization. **(D)** Kaplan-Meier curves showing progress free interval (PFI) of patients with pancreatic adenocarcinoma according to CITED4 expression (left) or CIRBP expression (right). Grouping via P-value minimization. **(E)** Feature plots showing the normalized expression of CITED4 in cell atlas related to Figure 1B. **(F)** Boxplot showing the CIBERSORT scores of immune cell subsets in CITED4 high- and low-expression groups. **(G)** Feature plots showing the ductal cells were stratified into CITED4-positive and CITED4-negative groups based on the expression status of CITED4 in ductal cells related to Figure 2A. **(H)** Box plots showing ICD scores for

CITED4-positive and CITED4-negative groups. **(I)** Boxplot showing the expression of ICOSLG and VTCN1 in high- and low-risk groups related to Figure 5L. **(J)** Scatter plots showing the significant negative spearman correlation of the expression of CITED4 with ICOSLG in the TCGA dataset. **(K)** Scatter plots showing the significant positive spearman correlation of the expression of CITED4 with VTCN1 in the TCGA dataset.
